# Kinetic Multi-omic Analysis of Responses to SARS-CoV-2 Infection in a Model of Severe COVID-19

**DOI:** 10.1128/JVI.01010-21

**Published:** 2021-09-27

**Authors:** Angelene M. Cantwell, Harinder Singh, Maryann Platt, Yanbao Yu, Yi-Han Lin, Yuji Ikeno, Gene Hubbard, Yan Xiang, Norberto Gonzalez-Juarbe, Peter H. Dube

**Affiliations:** a Department of Microbiology, Immunology, and Molecular Genetics, UT-Health San Antonio, San Antonio, Texas, USA; b Infectious Disease and Genomic Medicine Group, J. Craig Venter Institute, Rockville, Maryland, USA; c Barshop Institute for Longevity and Aging Studies, UT-Health San Antonio, San Antonio, Texas, USA; d Department of Pathology, UT-Health San Antonio, San Antonio, Texas, USA; e Select Agent Research Core, UT-Health San Antonio, San Antonio, Texas, USA; Loyola University Chicago

**Keywords:** SARS-CoV-2, COVID-19, proteomics, phosphoproteomics, RNA-seq, hamster

## Abstract

The host response to severe acute respiratory syndrome coronavirus 2 (SARS-CoV-2) is poorly understood due to a lack of an animal model that recapitulates severe human disease. Here, we report a Syrian hamster model that develops progressive lethal pulmonary disease that closely mimics severe coronavirus disease 2019 (COVID-19). We evaluated host responses using a multi-omic, multiorgan approach to define proteome, phosphoproteome, and transcriptome changes. These data revealed both type I and type II interferon-stimulated gene and protein expression along with a progressive increase in chemokines, monocytes, and neutrophil-associated molecules throughout the course of infection that peaked in the later time points correlating with a rapidly developing diffuse alveolar destruction and pneumonia that persisted in the absence of active viral infection. Extrapulmonary proteome and phosphoproteome remodeling was detected in the heart and kidneys following viral infection. Together, our results provide a kinetic overview of multiorgan host responses to severe SARS-CoV-2 infection *in vivo*.

**IMPORTANCE** The current pandemic caused by severe acute respiratory syndrome coronavirus 2 (SARS-CoV-2) infection has created an urgent need to understand the pathogenesis of this infection. These efforts have been impaired by the lack of animal models that recapitulate severe coronavirus disease 2019 (COVID-19). Here, we report a hamster model that develops severe COVID-19-like disease following infection with human isolates of SARS-CoV-2. To better understand pathogenesis, we evaluated changes in gene transcription and protein expression over the course of infection to provide an integrated multiorgan kinetic analysis of the host response to infection. These data reveal a dynamic innate immune response to infection and corresponding immune pathologies consistent with severe human disease. Altogether, this model will be useful for understanding the pathogenesis of severe COVID-19 and for testing interventions.

## INTRODUCTION

Severe acute respiratory syndrome coronavirus 2 (SARS-CoV-2) is a novel, highly contagious, β-coronavirus responsible for human coronavirus disease 2019 (COVID-19). Coronaviruses are large, enveloped, single-stranded RNA viruses that cause a variety of human and veterinary diseases (1). Since 2003, emerging zoonotic infections with coronaviruses have caused significant morbidity, mortality, and economic loss. In late 2019, human SARS-CoV-2 infections emerged in Wuhan, China, prior to spreading worldwide (1). In March 2020, the World Health Organization classified it as a pandemic. SARS-CoV-2 is very closely related to the two other recently emerged human-pathogenic β-coronaviruses (SARS-CoV and Middle East respiratory syndrome [MERS]-CoV). Unlike SARS-CoV-2, neither SARS-CoV nor MERS-CoV caused widespread disease in human populations.

SARS-CoV-2 is currently causing a spectrum of disease from asymptomatic carriage to severe manifestations, including death (2–6). Disease severity is linked to age, sex, and various comorbid conditions (7). The case fatality rate (CFR) varies based on geopolitical factors, including location, rate of testing, health care infrastructure, and containment/mitigation measures. The CFR has ranged from a global average of 1.4% to as high as 20% in people over 70 in Italy (7). The fact that the human population is naive to this virus and there are no specific curative treatments for COVID-19 emphasizes the urgent need for a detailed understanding of the pathogenesis of SARS-CoV-2.

Unlike SARS and MERS, patients infected with SARS-CoV-2 have high viral burdens prior to the onset of symptoms (8, 9). With an average of 5.2 days to the onset of symptoms, patients can develop symptoms up to 14 days after infection (9). Clinically, COVID-19 can be described in three stages (10), as follows: stage I is the viral infection stage, stage II is the pulmonary stage, and stage III is the hyperinflammation phase. The pulmonary stage is further divided into stages IIA and IIB that are delineated by the presence of pneumonia in stage IIB. In general, viral burdens decrease after the onset of symptoms, and there is a delay in the transition to the hyperinflammatory phase of the disease that correlates with worse outcomes. While these stages of disease are mainly a useful guide for clinical interventions, they also point to potential changes in the immune response to infection as disease progresses that likely drives COVID-19 pathogenesis.

Like other human-pathogenic β-coronaviruses, SARS-CoV-2 utilizes its surface-exposed spike protein to enter human cells. The spike protein receptor binding domain (RBD) of the SARS-CoV-2 virus interacts with the human ACE2 protein to facilitate virus uptake (11). The SARS-CoV-2 spike protein does not bind efficiently to the mouse ACE2 protein, but it will infect other experimental animal models, including ferrets, hamsters, and certain species of nonhuman primates (NHP) (12–15). COVID-19-like disease in hamsters and certain NHPs can mimic moderate disease severity in humans (12, 13, 15). The novelty of SARS-CoV-2 is reflected in the lack of information available about the host response to infection and the subsequent hyperinflammatory responses. Our understanding of SARS-CoV-2 pathogenesis has been impacted by the lack of an animal model that accurately mimics severe human disease. While studies of SARS-CoV and MERS-CoV provide some insight into pathogenesis (16), SARS-CoV-2 has a different initial clinical presentation suggesting differences in host-pathogen interactions.

The innate host response to infection is initiated when the presence of pathogen components is detected by pathogen recognition receptors (PRRs) (17). PRRs recognize conserved structural features of pathogens known as pathogen-associated molecular pattern molecules (PAMPs), such as nucleic acids and cell wall components (18). The single-stranded RNA genome and double-stranded replication intermediates of SARS-CoV-2 could be recognized by several PRRs, including Toll-like receptors (TLRs) (19, 20) or cytosolic nucleic acid receptors (21). The recognition of PAMPs by PRRs initiates activation of signaling pathways that induce the expression of inflammatory genes and, in the case of β-coronaviruses, an antiviral response. Early innate antiviral responses are mediated by interferons with the classical responses initiated by type I interferon (IFN-α/β) and type III interferon (IFN-λ) (22). Interferons induce the expression of numerous interferon-stimulated genes (ISG) that impair viral replication. Additionally, type II interferon (IFN-γ) plays an important role in activating the immune system and polarizing the adaptive immune response to viruses (23). Currently, the exact PRRs that recognize SARS-CoV-2 and the subsequent nature of the interferon response and ISGs induced during the course of SARS-CoV-2 infection remain largely unknown. PRR activation also induces a strong inflammatory response by modulating the expression of cytokines, chemokines, and other immune molecules from innate immune cells. Although it is clear from clinical reports and limited studies in experimental animals that severe SARS-CoV-2 infection induces a strong inflammatory response, the signaling pathways activated remain mostly poorly defined.

Initial studies of the transcriptional responses to SARS-CoV-2 infection demonstrated a strong inflammatory cytokine response and a blunted interferon response relative to other respiratory viruses (24, 25). While several other studies have reported changes in the transcriptome following infection of cells in culture (24, 25), little is known about global kinetic changes in gene and protein expression during *in vivo* models of infection. In contrast, single-cell transcriptomic analysis of PBMCs from patients with varying severities of COVID-19 demonstrated strong interferon and inflammatory gene signatures associated with severe disease (26). Specifically, Lee and colleagues reported that type I interferon and tumor necrosis factor alpha (TNF-α)/IL-1β correlated with COVID-19 severity (26).

How the proteome of target organs change during the development of acute COVID-19 is not understood. Recently, changes in the serum proteome and metabolome from COVID-19 patients defined signatures that correlated with disease severity (27). Likewise, a proteomic analysis of SARS-CoV-2-infected Caco-2 cells revealed global changes in the cellular proteome and potential targets for therapeutic intervention (28). To date, a kinetic global analysis of lung-specific changes in the proteome is lacking. Moreover, how SARS-CoV-2 infection impacts cellular signaling during the development of acute disease in target organs is not defined. Cellular signaling is often mediated by phosphorylation, and a recent analysis of the phosphoproteome of Vero E6 cells identified a variety of kinases impacted by infection (29). However, changes in the phosphoproteome of SARS-CoV-2-infected tissues are currently unknown. Here, using a small-animal model of severe COVID-19-like disease, we report global integrated kinetic changes in the transcriptome, proteome, and phosphoproteome of the lung and multiproteomic changes in hearts and kidneys in the development of acute COVID-19-like disease in the hamster.

## RESULTS

### Intratracheal infection of hamsters causes a severe COVID-19-like disease.

**FIG 1:**
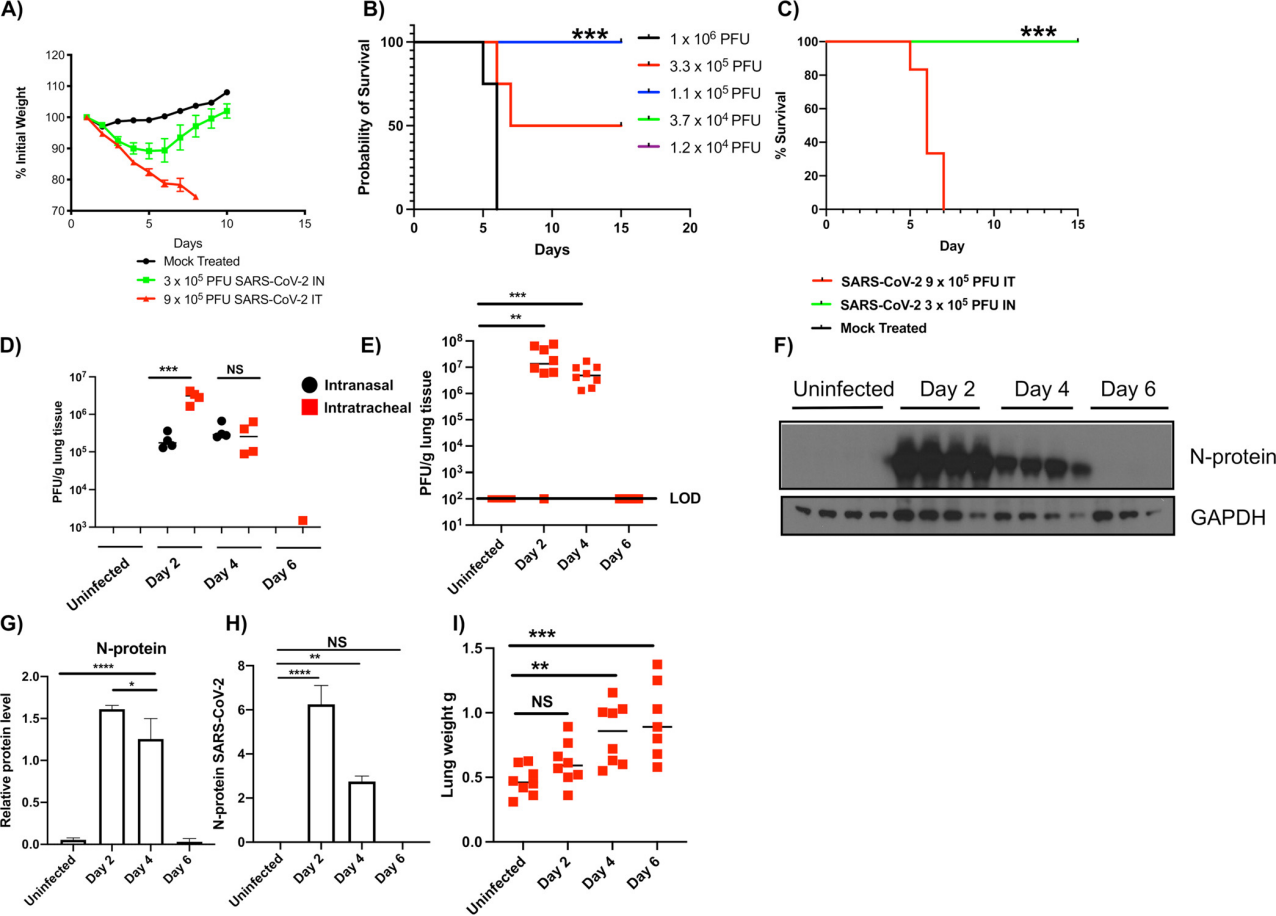
Intratracheal SARS-CoV-2 infection in golden Syrian hamsters leads to severe pulmonary inflammation that mimics human responses. Male 5- to 6-week-old golden Syrian hamsters were intranasally or intratracheally infected with SARS-CoV-2 isolate USA-WA-1/2020 or mock challenged with vehicle (DMEM), and lungs were excised at days 2, 4, and 6 postinfection. (A) Percent weight change. (B) LD_50_ analysis (note green, purple, and blue lines denoting 100% survival are superimposed). (C) Survival. (D) Lung viral burden measured via plaque assays following infection with 3 × 10^5^ PFU; black symbols, intranasal; red symbols, intratracheal. (E) Lung viral burden measured via plaque assays following i.t. infection with 9 × 10^5^ PFU (LOD, level of detection). (F) SARS-CoV-2 nucleoprotein immunoblots. (G) Relative quantitation of results in F. (H) Mass spectrometry analysis targeting SARS-CoV-2 nucleoprotein. (I) Wet lung weight; NS, not significant. (B, C) ***, *P* = 0.0005; log-rank analysis. (D to I) *, *P* = 0.05; **, *P* = 0.005; ***, *P* = 0.0005; ****, *P* = 0.00005; analysis of variance (ANOVA) with multiple comparisons posttest. *n* = 8 to 10 (A, C), 3 to 4 (B, D to G), and 7 to 8 (I).

Infection of humans with SARS-CoV-2 causes a spectrum of disease ranging from asymptomatic carriage to severe pneumonia and death (2). Previous reports suggested that intranasal (i.n.) infection of hamsters with SARS-CoV-2 led to mild-to-moderate lung disease (12, 30). Consistent with the findings of others, i.n. infection of hamsters with 3 × 10^5^ PFU of SARS-CoV-2 led to ∼12% maximal body weight loss, but all animals recovered (Fig. 1A). These data suggest that i.n. infection of hamsters with SARS-CoV-2 induces a moderate infection sufficient to induce morbidity, as evidenced by weight loss. However, i.n. infection is limited by the volume and titer of virus that can be instilled and intratracheal (i.t.) infection allows for higher titer virus challenge. To model severe COVID-19-like disease, we further characterized i.t. infection of the hamster. We determined the i.t. 50% lethal dose (LD_50_) of isolate USA-WA-1/2020 to be 3 × 10^5^ PFU (Fig. 1B) with hamsters infected with 9 × 10^5^ PFU i.t. succumbing by day 7 (Fig. 1C). We tested if i.t. infection impacted viral burdens in the lungs. Briefly, hamsters were either mock infected (day 0) or infected i.n. or i.t. with 3 × 10^5^ PFU of SARS-CoV-2, and viral burdens were determined in the lungs on days 0, 2, 4, and 6 (Fig. 1D). On day 2, hamsters infected i.t. had significantly higher viral burdens than those infected i.n., and viral burdens equalized on day 4. These data suggest that i.t. infection results in an increased viral burden early during infection compared with i.n.-infected hamsters.

To model severe COVID-19-like disease, hamsters were infected intratracheally (i.t.) with 9 × 10^5^ PFU of SARS-CoV-2, USA-WA-1/2020. This procedure led to rapid and progressive weight loss resulting ultimately in death on days 5 to 7 postinfection (Fig. 1A and C). High titers of replicating virus were detected in the lungs at days 2 and 4 after infection with a mean viral burden of 1.3 × 10^7^ and 4.9 × 10^6^ PFU/g lung tissue, respectively (Fig. 1E). No replicating virus was detected in lung tissues on day 6 after infection. The presence of SARS-CoV-2 was confirmed in lung tissues using immunoblotting for the SARS-CoV-2 N protein (Fig. 1F and G). Additionally, the N protein was detected in hamster lungs using mass spectrometry (Fig. 1H). N protein was readily detected in lung extracts on days 2 and 4 postinfection but not on day 6, consistent with the lack of detectable virus at this time point.

**FIG 2:**
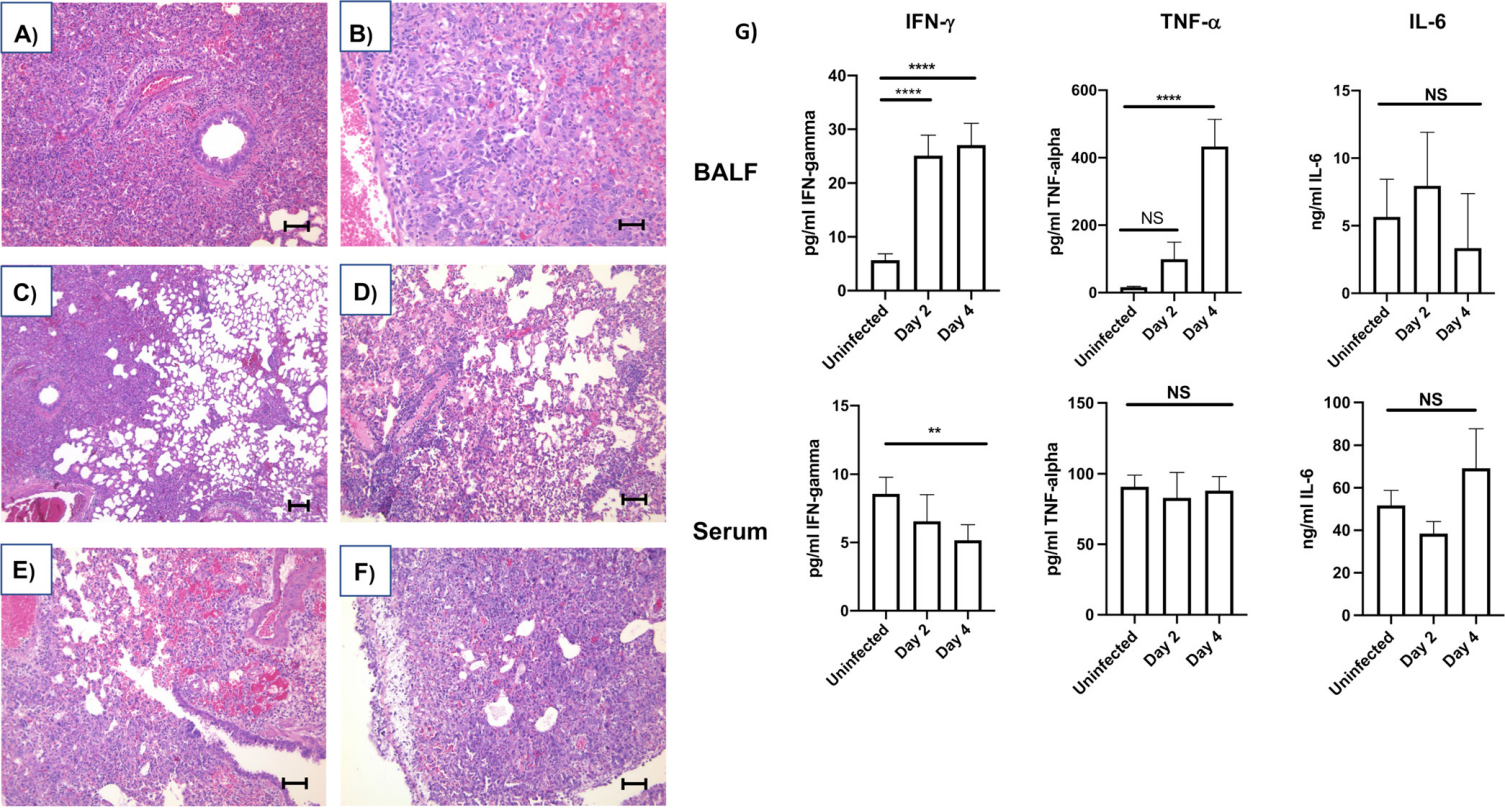
Inflammation of the SARS-CoV-2-infected hamster lung. Male 5- to 6-week-old golden Syrian hamsters were intratracheally infected with SARS-CoV-2 isolate USA-WA-1/2020 or mock challenged with vehicle (PBS), and lungs were excised at days 2, 4, and 6 postinfection. (A and B) Hematoxylin and eosin staining of lung sections on days 2 and 6 postinfection, respectively (A, 100×, scale bar is 100 mm; B, 200×, scale bar is 50 mm). (C) Histology with low magnification shows the mixture of dilated air space in alveoli (right side) and solid part (left side) filled with mononuclear inflammatory cells in intra-alveolar space, alveolar septa, and peribronchial and perivascular areas. Day 2 postinfection, H&E, 40×. (D) Pulmonary edema and pneumocyte desquamation with inflammatory cells infiltration were commonly observed. Dilated alveoli, perivasculitis, and thickening of alveolar septa with inflammatory cells infiltration were also presented. Some cases showed the mononuclear cell aggregation and adherence to endothelial cells in the vessels. Day 4 postinfection, H&E, 100×. (E) Different degrees of congestion were commonly observed, and intra-alveolar hemorrhage was often associated in the area where prominent congestion occurred. Bronchial epithelial hyperplasia, perivascular inflammation and edema, organized pneumonia-like lesion with inflammatory cells, and giant cells were also presented. Day 6 postinfection, H&E, 100×. (F) Reactive mesothelial hyperplasia with inflammatory cell infiltration and subpleural edema were observed in the advanced stages. Alveolar space and septa were filled by inflammatory cells and giant cells with interstitial thickening, which resembles organized pneumonia. Day 6 postinfection, H&E, 100×. (G) Inflammatory cytokine expression. ELISA determined the concentration of IFN-γ, TNF-α, and interleukin-6 (IL-6) in the BALF or serum of uninfected controls or hamsters infected i.t. with 9 × 10^5^ PFU SARS-CoV-2 for 2 or 4 days. Data were evaluated by Kruskal-Wallis test with Dunn’s multiple-comparison posttest. Asterisks denote the level of significance observed, as follows: **, *P* ≤ 0.005; ****, *P* ≤ 0.00005. *n*= 6 hamsters per time point.

We observed that i.t. infection caused progressive changes in inflammatory histopathology culminating with findings consistent with acute viral pneumonia and autopsy findings reported for patients that died from COVID-19 (31, 32). Hamsters were challenged with SARS-CoV-2; animals were euthanized on days 2, 4, and 6 after infection; and lung pathology was evaluated. Tissues were compared with mock-infected hamster lungs. Gross examination revealed inflamed lungs on days 2, 4, and 6 postinfection. Gross indications of pulmonary inflammation included progressive increase in lung firmness, areas of hemorrhage, and lobular red hepatization in all animals on days 4 and 6 (data not shown). The gross weight of the lungs was significantly increased on days 4 and 6 after infection that is consistent with edema and acute inflammatory reaction (Fig. 2A). Microscopic examination of lungs showed dilated airspaces and mononuclear inflammatory cell infiltration (predominantly lymphocytes and macrophages) in bronchial walls, alveolar septa, and perivascular area evident on days 2 and 4 postinfection (Fig. 2C and D). These inflammatory changes were accompanied with congestion and edema (intra-alveolar space, perivascular, and subpleural area) and type 2 pneumocyte desquamation present as early as day 2 and progressing in severity to day 6 (Fig. 2A and E). In the advanced stage, at day 6 postinfection, type 2 pneumocyte hyperplasia and severe inflammatory cell infiltration with mild interstitial fibrotic changes in the alveolar wall became more prominent, which lead to interstitial thickening. Type 2 pneumocytes showed enlarged cytoplasms with a large nucleus and a prominent nucleolus, which has morphologic features similar to nuclear inclusion, and multinucleated giant cells are also present (Fig. 2B and F). The severity of these morphological changes became more pronounced with time. Bronchial epithelium also became hyperplastic, and acute inflammatory exudates (with neutrophils) in bronchus were observed in some cases. In the far advanced stage, close to end stage, intra-alveolar organization of exudates occurred along with the infiltration of type 2 pneumocytes and other inflammatory cells (predominantly lymphocytes and macrophages) in the majority of the alveolar space. These changes in alveolar space were accompanied with prominent hyperplasia of the bronchial epithelium, severe interstitial inflammatory cell infiltration, and mild fibrotic changes and began to resemble organizing pneumonia (Fig. 2F). These data correlated with increases in inflammatory cytokines in the lung (Fig. 2G). The robust infiltration of neutrophils and macrophages late in infection, at day 6 postinfection, in the absence of viral replication, is consistent with the hyperinflammatory stage of human disease (Fig. 2A and B). Altogether, these data show the development of severe pulmonary pathology consistent with COVID-19-like disease in the hamster following SARS-CoV-2 infection.

### Respiratory infection of hamsters with SARS-CoV-2 causes global changes in pulmonary gene and protein expression.

Hamsters infected with SARS-CoV-2 develop severe respiratory disease, and yet the changes in gene and protein expression following infection are poorly defined. To further interrogate host responses in the development of severe COVID-19-like disease, we characterized global changes in the expression of lung mRNA, protein, and phosphoproteins during the development of severe COVID-19-like disease in the hamster.

### Pulmonary infection with SARS-CoV-2 leads to global changes in the transcriptome.

**FIG 3:**
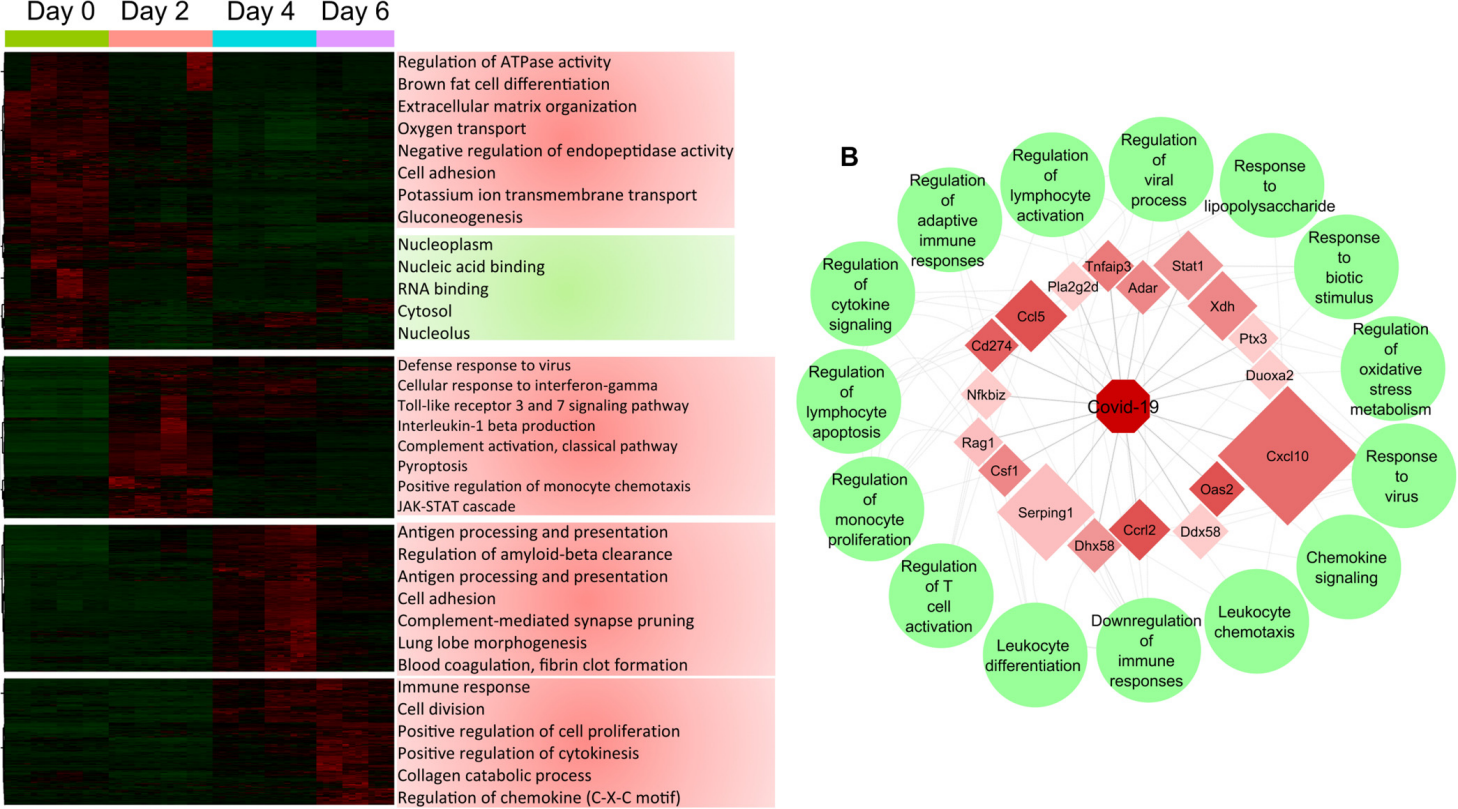
Transcriptomic analysis of hamster lungs reveals kinetic modulation of host responses after SARS-CoV-2 infection. Male 5- to 6-week-old golden Syrian hamsters were infected intratracheally with SARS-CoV-2 isolate USA-WA-1/2020 or mock challenged with vehicle (PBS), and lungs were excised at days 2, 4, and 6 postinfection. (A) Heatmap of RNA-seq expression changes and main gene ontology terms for each cluster. (B) Correlation plot showing highly correlated transcripts with SARS-CoV-2 genome abundance. The size of a transcript represents the relative abundance, and the color indicates the fold change of the transcript between day 0 and day 2 with the associated GO terms in green circles.

**FIG 4:**
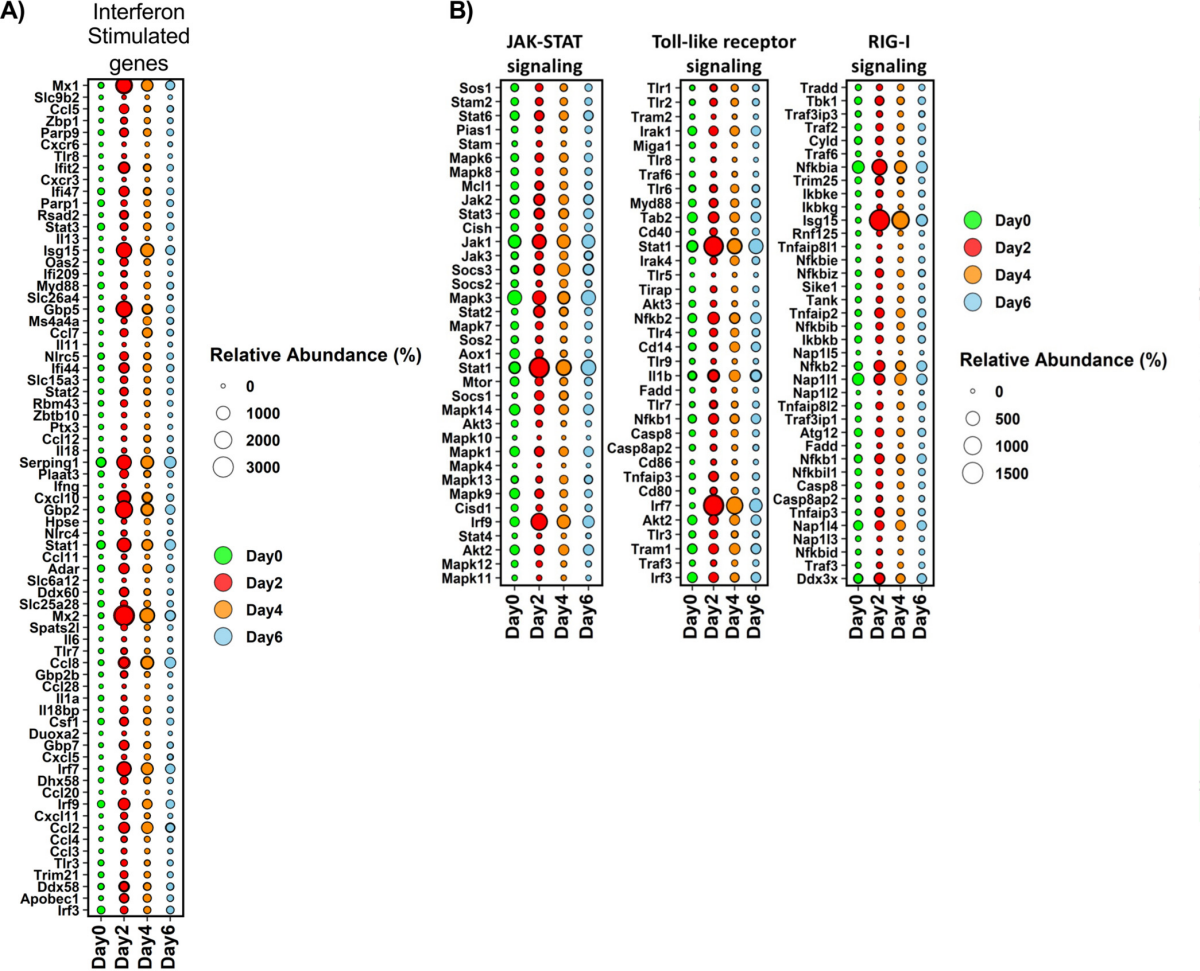
Interferon-stimulated genes and innate immune signaling pathways are kinetically upregulated upon severe SARS-CoV-2 infection. (A) Bubble plot of significantly changed transcripts associated with interferon responses. Bubble size denotes relative abundance, and the following colors denote time points: green (day 0, uninfected), red (day 2), orange (day 4), and blue (day 6). (B) Bubble plot of significantly changed transcripts associated with JAK-STAT, Toll-like receptor, and RIG-I signaling pathways. Bubble size denotes relative abundance, and the following colors indicate time points: green (day 0, uninfected), red (day 2), orange (day 4), and blue (day 6).

The transcriptome was evaluated by transcriptome sequencing (RNA-seq) uninfected lungs and lungs infected with 9 × 10^5^ PFU on days 2, 4, and 6 postinfection. Infection resulted in the differential expression of 14,345 transcripts with at least a log_2_ fold change of ≥2.0 and *P* value of ≤ 0.05 across the course of infection. Hierarchical clustering and gene ontogeny (GO) analysis of differentially expressed transcripts revealed 4 distinct clusters of differentially expressed transcripts correlating with temporal changes in the host response to infection. On day 2 postinfection, there is an increase in transcripts associated with GO terms encompassing antiviral responses and immunity to infection (Fig. 3A). Both type I and II interferon stimulated gene (ISG) signatures were readily apparent in the transcriptome on day 2 postinfection, and these responses remained dominant through the course of infection (Fig. 4A). Type III interferon induces patterns of gene expression that are similar to type I interferon (22). Indeed, type III interferon responses have been reported following SARS-CoV-2 infection in other systems (33). At this time, genes for type III interferon have not been identified in the Syrian hamster. However, we cannot rule out that the type I ISG signature is not a mixed response to type I and III interferons, especially on days 4 and 6 postinfection.

**FIG 5:**
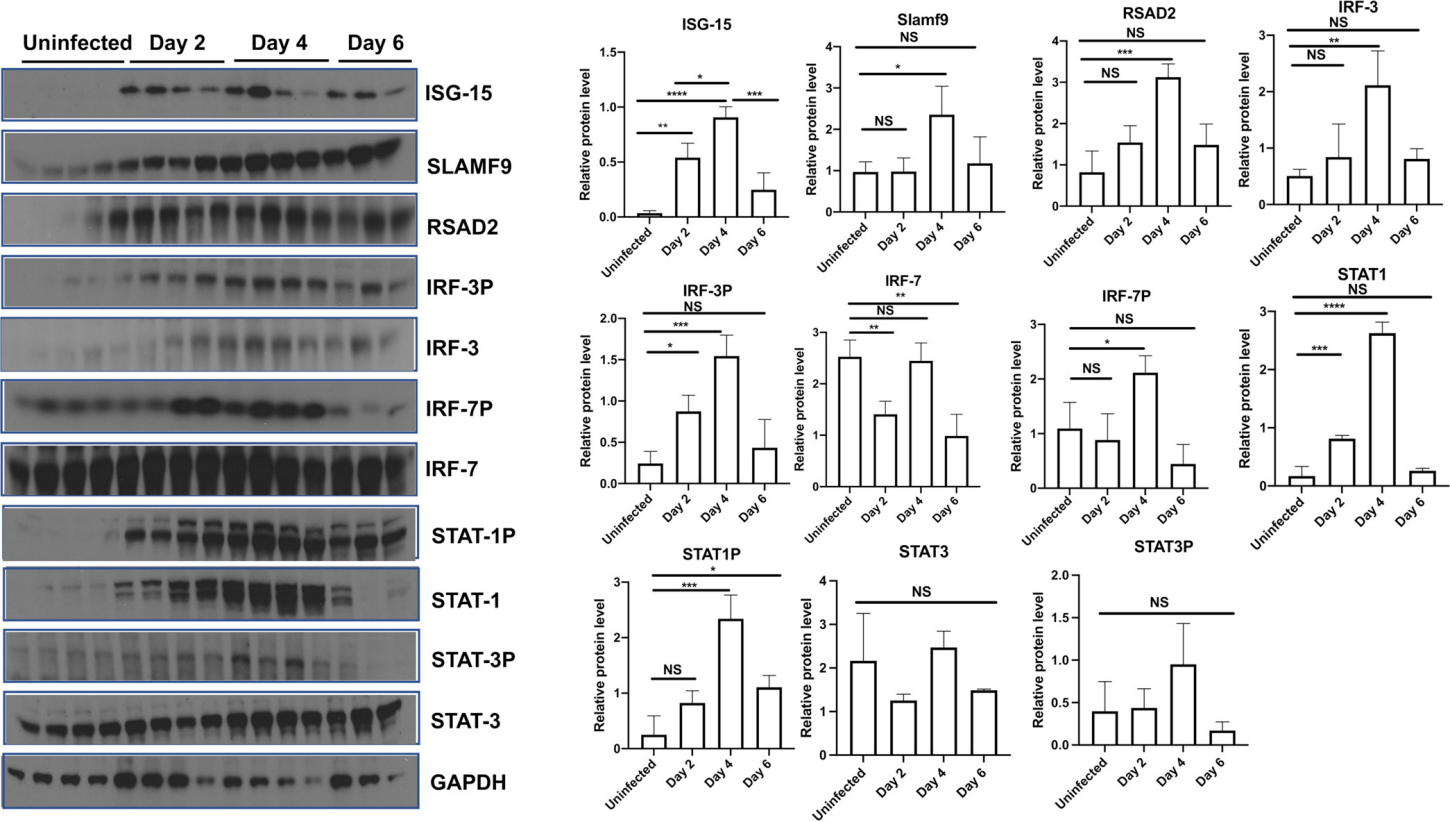
Severe SARS-CoV-2 infection leads to interferon-stimulated antiviral proteins. Male 5- to 6-week-old golden Syrian hamsters were infected intratracheally with SARS-CoV-2 isolate USA-WA-1/2020 or mock challenged with vehicle (PBS), and lungs were excised at days 2, 4, and 6 postinfection. Immunoblots and relative protein level (densitometry) against ISG-15, SLAMF9, RSAD2, IRF-3, IRF-3p, IRF-7, IRF-7p, STAT1, STAT1p, STAT3, and STAT3p. Kruskal-Wallis test with Dunn’s multiple-comparison posttest. Asterisks denote the level of significance observed, as follows: *, *P* ≤ 0.05; **, *P* ≤ 0.01; ***, *P* ≤ 0.001; ****, *P* ≤ 0.0001.

Plots of the resulting average levels of marker gene expression for the RIG-I (34), JAK-STAT (35), and Toll-like receptor (36) pathways further showed temporal changes to antiviral responses shown as bubble charts (Fig. 4B). As expected, there was a strong induction of transcripts associated with antiviral responses mediated via interferon and TLR signaling which correlated with the temporal change in the expression of interferon-stimulated transcription factors, including phosphorylated IRF-3/7 and STAT1/3 (Fig. 5). These data associated with the temporal expression of interferon-stimulated antiviral proteins ISG-15, SLAMf9, and RSAD2 (Fig. 5). Consistent with the histopathological analysis of lung tissues, significant increases in transcripts associated with pyroptosis and monocyte recruitment increased on days 2 through 6, while transcripts associated with blood clotting/fibrin clot formation were observed later on days 4 and 6 postinfection. Moreover, temporal changes in the expression of genes associated with cell death pathways were readily observed in the transcriptional analysis (Fig. 3 and 4). Altogether, these data reveal large changes in transcription following SARS-CoV-2 challenge that were associated with host response to infection and detrimental physiological changes in the pulmonary compartment.

### SARS-CoV-2 transcripts correlate with host transcripts associated with regulation of both antiviral and other immune responses.

As shown in Fig. 1D and E, peak viral replication in the lung was observed on day 2 following infection, and at this time point, viral RNA (transcripts and genomes) and proteins were readily detectable in the lungs of infected animals (Fig. 1F to H and data not shown). Host responses correlating to viral genomes were determined and are represented in a correlation plot (Fig. 3B). There was a robust correlation between SARS-CoV-2 transcripts and the IFN-γ-induced chemokine Cxcl-10 (IP-10) and to a lesser extent Csf1, Ccrl2, and Ccl5, which are all involved in the recruitment of immune cells to the site of infection. Viral genomes strongly correlated with expression antiviral genes (Csf1, Oas2, Ddx58, Dhx58, Serping1, Pla2g2d, Adar, Stat1, and Ptx3), as well as genes involved in the regulation of inflammation (CD274, Tnfaip3, and Nfkbiz). Among the host genes correlating with viral transcripts, most were associated with either IFN signaling or TNF-α signaling. Consistent with these findings, concentrations of these cytokines were increased significantly in the bronchoalveolar lavage fluid (BALF) of infected hamsters on day 2 (IFN-γ) and day 4 (IFN-γ and TNF-α) postinfection (Fig. 2G).

### SARS-CoV-2 infection results in temporal changes to the lung proteome.

**FIG 6:**
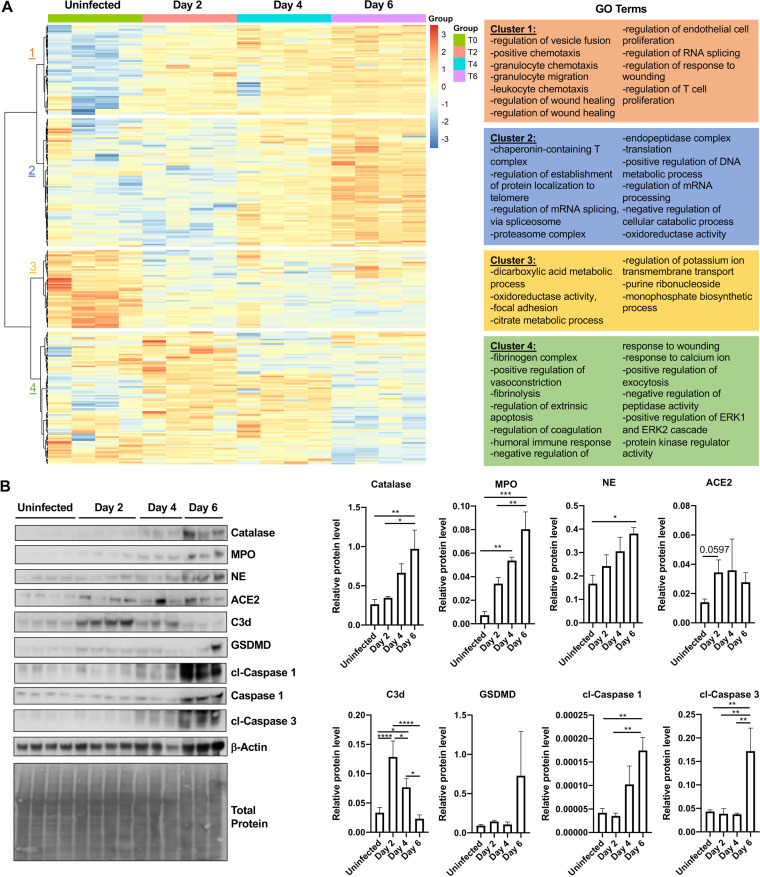
Severe SARS-CoV-2 infection leads to lung proteome remodeling. Male 5- to 6-week-old golden Syrian hamsters were infected intratracheally with SARS-CoV-2 isolate USA-WA-1/2020 or mock challenged with vehicle (DMEM), and lungs were excised at days 2, 4, and 6 postinfection. (A) Proteomic changes of hamster lungs after SARS-CoV-2 infection or mock challenge (*n* = 3 to 4 per time point). Hierarchical clustering of label-free quantification (LFQ) intensities of significantly changed proteins (ANOVA, *P* < 0.05) revealed four distinct clusters. Their abundance profiles among the groups were plotted in the heatmap. Enriched GO biological process terms are indicated for each marked cluster. (B) Immunoblots for complement C3b (C3b), myeloperoxidase (MPO), catalase, neutrophil elastase (NE), gasdermin D (GSDMD), caspase-1, cleaved caspase-1, cleaved caspase-3, and actin (*n* = 3 to 4 per group). Total protein is also shown as a loading control. Histograms of protein level quantification (densitometry). Kruskal-Wallis test with Dunn’s multiple-comparison posttest. Asterisks denote the level of significance observed, as follows: *, *P* ≤ 0.05; **, *P* ≤ 0.01; ***, *P* ≤ 0.001; ****, *P* ≤ 0.0001.

To evaluate changes in the lung proteome following SARS-CoV-2 infection, we utilized label-free quantitative proteome analysis of lungs on days 2, 4, and 6 after infection relative to controls. A total of 769 proteins were expressed differentially (*P* ≤ 0.05) with a minimum of 2 peptides/protein. Hierarchical clustering revealed four distinct clusters of proteins that changed expression over the course of infection. Evaluation of the biological processes (GO terms) associated with cluster 1 differentially expressed proteins revealed a progressive increase of proteins involved in chemotaxis, RNA splicing, leukocyte chemotaxis, granulocyte migration, and regulation of T cell proliferation (cluster 1, Fig. 6A). In cluster 2, proteins showed low expression in uninfected hamsters and at day 2 with a stark increase on days 4 and 6 after infection. They included GO terms for endopeptidase activity, mRNA processing, protein localization to telomeres, and oxidoreductase activity (cluster 2, Fig. 6A). Proteins in cluster 3 revealed a progressive decrease in proteins involved in metabolism and homeostasis relative to uninfected controls (cluster 3, Fig. 6A). Finally, there were increases in distinct clusters of proteins associated with immune responses and kinase activity function on days 2 and 4 that decreased by day 6 postinfection. They included GO terms for ERK1 and ERK2 cascades, cell death, vasoconstriction, calcium changes, and regulation of humoral immune responses (cluster 4, Fig. 6A). These data are consistent with the robust infiltration of inflammatory cells in lung tissues observed in histological sections of SARS-CoV-2-infected hamsters at these time points. Hallmarks of cell death and inflammation, including cleaved caspase-1 and −3, gasdermin-D, neutrophil elastase, catalase, complement C3, and myeloperoxidase were validated in lung tissues on day 6 after infection via immunoblot (Fig. 6B). Importantly, and consistent with the proteomic analysis, significant increases in neutrophil elastase, myeloperoxidase, and catalase were observed on days 2 and 4 after infection, peaking on day 6 relative to controls (Fig. 6B). Interestingly, the proteomic analysis revealed a role for neutrophils late in infection that was not apparent by the transcriptomic analysis (see Fig. S1 in the supplemental material).

**FIG 7:**
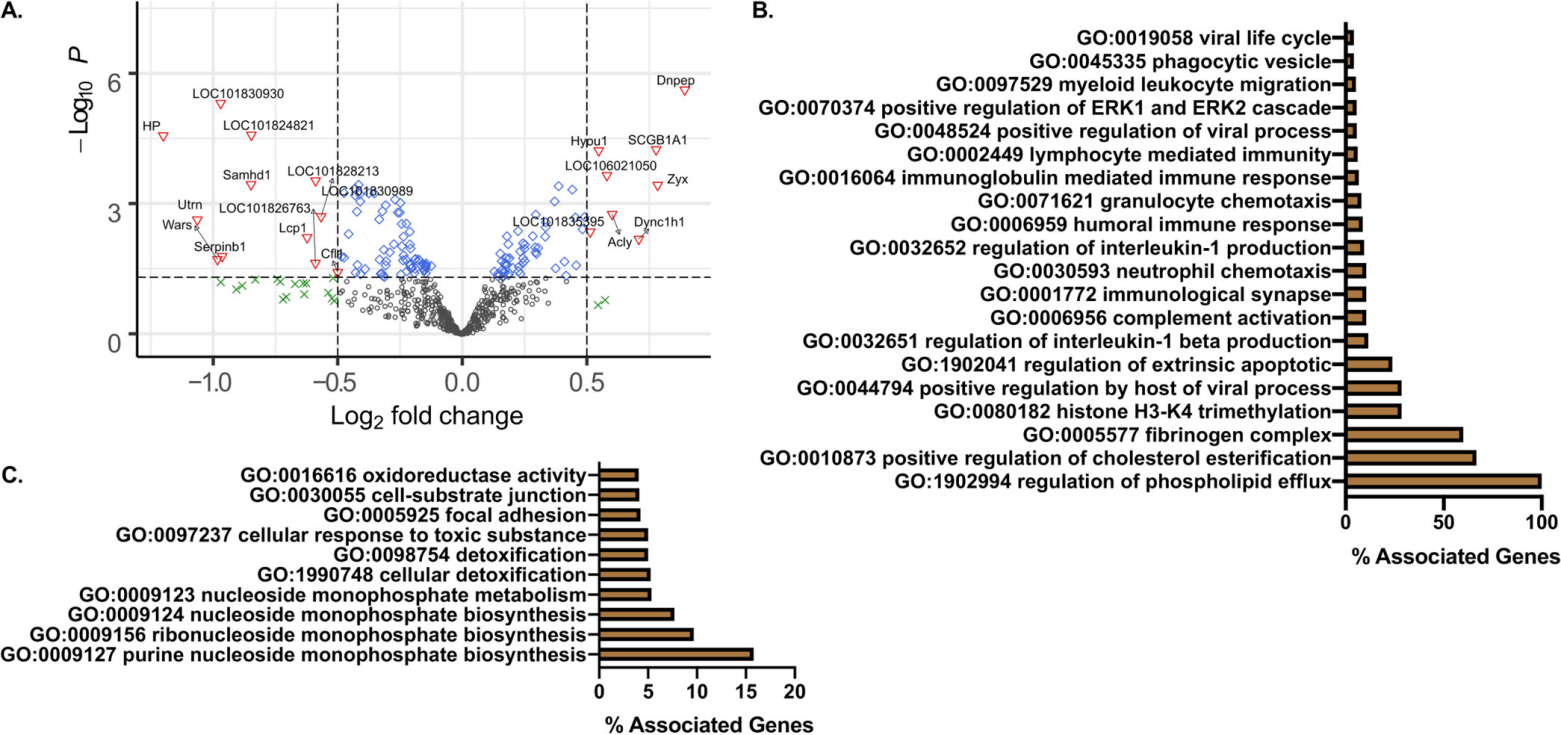
Proteomic analysis of early time point after infection shows strong innate immune responses against viral infection. (A) Quantitative comparison of SARS-CoV-2-infected or mock-infected (DMEM challenge) hamsters (*n* = 3 to 4 per group). Log_2_ fold change of the proteins (*x* axis) and their significance (*P* < 0.05; *y* axis) were plotted. Up- and downregulated proteins are highlighted in red and blue, respectively. (B) Percent associated genes of representative GO terms for upregulated proteins at day 2 postinfection relative to uninfected lungs. (C) Percent associated genes of representative GO terms for downregulated proteins at day 2 postinfection relative to uninfected lungs.

A further analysis of upregulated proteins on day 2 postinfection (Fig. 7A) revealed processes associated with IFN-γ and TNF-α signaling relative to uninfected lungs consistent with changes in pulmonary cytokines (Fig. 2G). In addition, GO terms associated with viral replication, phagocytosis, neutrophil activity, cholesterol changes, and cell death were upregulated (Fig. 7B). At the same time, significantly downregulated GO terms represented metabolic processes, oxidative responses, and cellular detoxification (Fig. 7C). Altogether, these data demonstrate a dynamic change in protein expression significantly correlated with the presence of SARS-CoV-2 genome transcripts.

### SARS-CoV-2 infection of the lungs leads to remodeling of the phosphoproteome.

**FIG 8:**
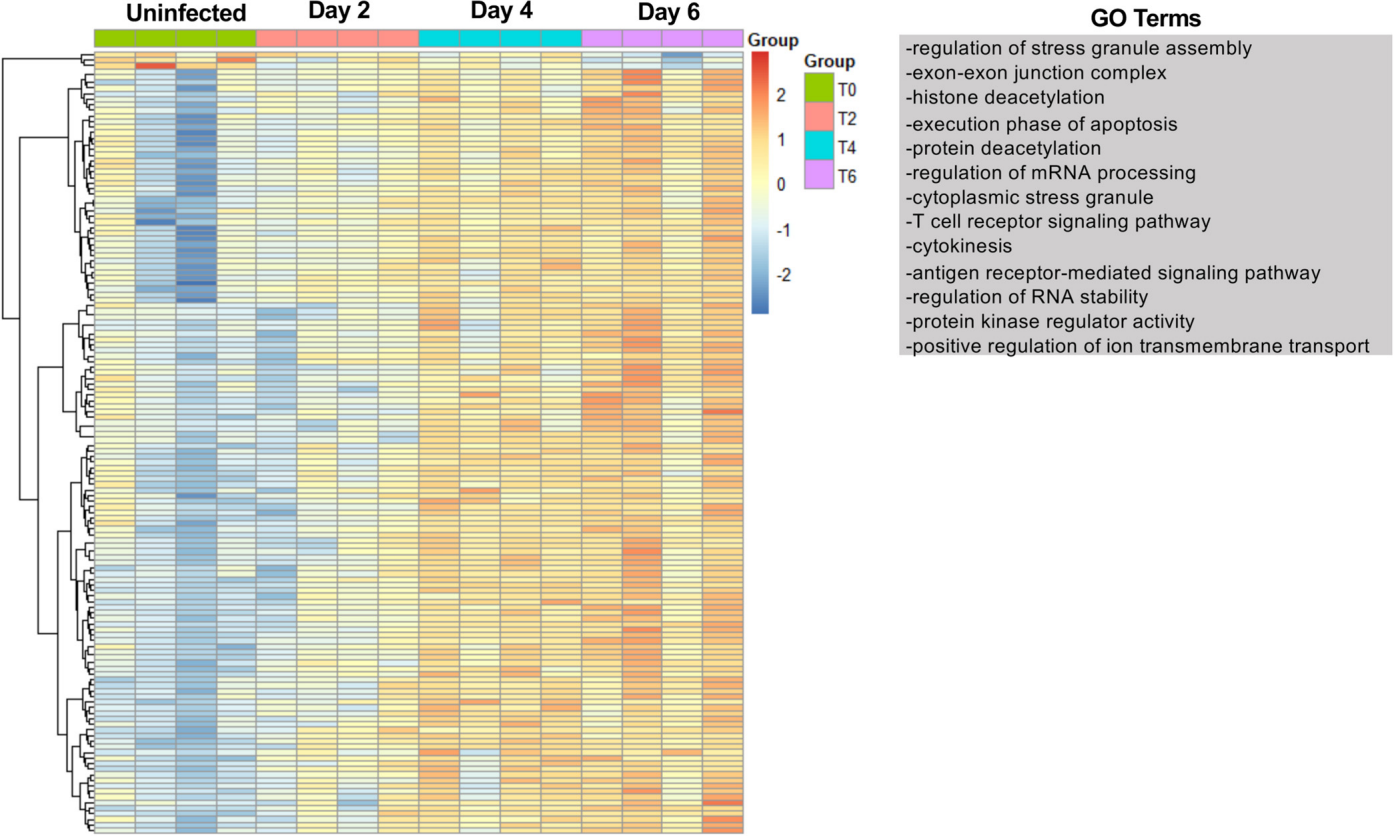
SARS-CoV-2 infection promotes lung phosphoproteome remodeling. Male 5- to 6-week-old golden Syrian hamsters were infected intratracheally with SARS-CoV-2 strain USA-WA-1/2020 or mock challenged with vehicle (DMEM), and lungs were excised at days 2, 4, and 6 postinfection. Heatmap of phosphorylation changes of hamster lung proteins after SARS-CoV-2 infection or mock challenge (*n* = 3 to 4 per time point). Hierarchical clustering of significantly changed phosphorylation state of proteins (ANOVA; *P* ≤ 0.05) revealed 2 distinct major clusters. Enriched GO biological process terms are indicated for phosphoproteins with increasing phosphorylation through the course of infection.

Analyses of the transcriptome and the proteome following SARS-CoV-2 infection demonstrate dynamic changes in the pulmonary environment from an early and consistent ISG expression to late expression of neutrophil-associated proteins. Cellular signaling is often mediated by phosphorylation, and to evaluate the impact of SARS-CoV-2 infection on the pulmonary phosphoproteome, we utilized TiO_2_ enrichment of phosphopeptides, followed by label-free liquid chromatography-tandem mass spectrometry (LC-MS/MS) analysis. A total of 184 proteins were identified as differentially phosphorylated (*P* ≤ 0.05). Hierarchical clustering revealed that the majority of proteins increased phosphorylation over time (Fig. 8). A small cluster of phosphoproteins had higher expression at time 0 and through day 4 postinfection that decreased on day 6. These phosphoproteins were VCL, SEPTIN4, Amph, Sptbn1, ACE2, Akap2, Pebp1, Exosc5, Slc16a1, Limch1, and Csrp1 that were associated with biological processes involved in cytoskeleton organization and cell junctions. The major cluster of phosphoproteins found progressively increased expression from time 0 to day 6. Phosphoproteins involved in histone deacetylation, cell death, T cell receptor signaling, and antigen receptor-mediated signaling were increased in the lungs of infected hamsters. GO term analysis of the phosphoproteome was consistent with analysis of the transcriptome and proteome through identification of increased expression of phosphopeptides involved in antiviral responses, cell death, and stress responses. However, phosphoproteome analysis also revealed an increase in biological processes associated with cytokinesis, protein kinase regulator activity, and mRNA processing (Fig. 8). All phosphoproteins and GO terms associated with the phosphoproteome are shown in Data Set S1 in the supplemental material. These processes were not readily apparent from the analysis of the transcriptome or the proteome.

### Integrated multi-omic analysis of lung responses during severe SARS-CoV-2 infection.

**FIG 9:**
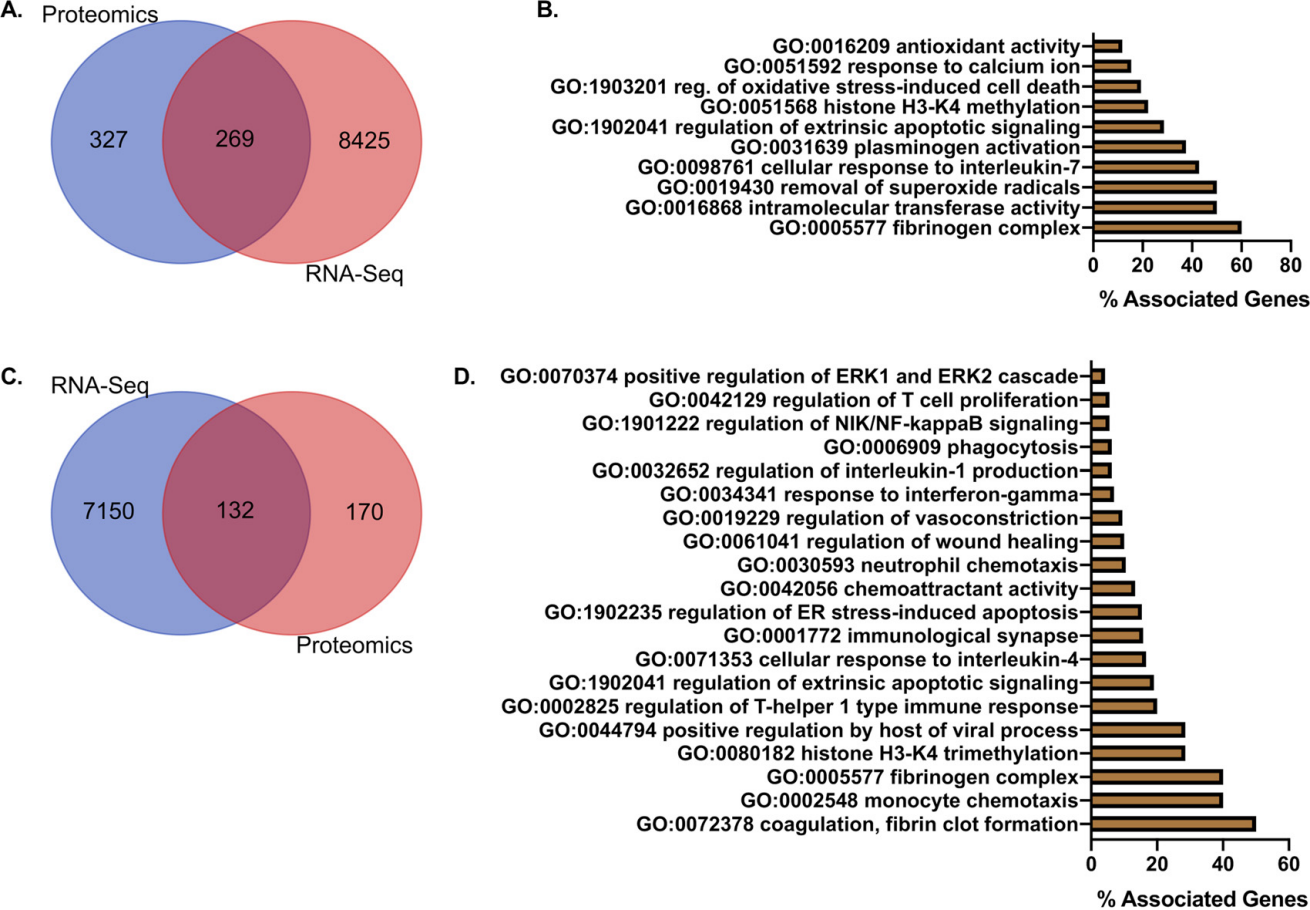
Integrated multi-omics analysis of lung transcriptome and proteome shows strong tissue injury and innate immune responses upon severe SARS-CoV-2 infection. (A) Venn diagram of transcripts with matching Uniprot IDs and global lung proteome. (B) Percent associated genes of representative GO terms for 269 globally shared factors in unfiltered proteome and transcriptome. (C) Venn diagram of transcripts with matching Uniprot IDs and global lung proteome factors significantly altered in at least one time point (0, 2, 4, and 6 days). (D) Percent associated genes of representative GO terms for 132 significantly changed shared factors in at least one time point (0, 2, 4, and 6 days).

An analysis of transcriptional factors with Uniprot identifiers (IDs) present in the global proteomic analysis showed 269 factors present in the lungs in both omic approaches (Fig. 9A). Selected GO analysis of the shared factors showed terms for genes associated with oxidative stress, cell death, cellular immune responses, and histone methylation (Fig. 9B). The full GO term list is presented in Data Set S2 in the supplemental material. A further analysis of significantly changed factors showed 132 common genes (Fig. 9C). GO analysis showed terms associated with cell death, neutrophil chemotaxis, phagocytosis, regulation of NIK/NF-κB signaling, viral replication, coagulation, endoplasmic reticulum (ER) stress, and immune cell responses (Fig. 9D). A complete list of shared host factors is shown in Data Set S2. These data confirm significant immune and cellular changes induced by SARS-CoV-2 infection through multi-omic approaches.

### Extrapulmonary SARS-CoV-2 infection results in changes to the proteome and phosphoproteome.

Severe COVID-19 is known to impact multiple organ systems, with cardiovascular and renal complications occurring frequently. A similar trend in viral titers to that observed in the lungs was also found in the hearts and kidneys of infected hamsters (see Fig. S2 in the supplemental material). Viral burdens in hearts and kidneys are reduced substantially compared with lung burdens (Fig. 1C). Although viral burdens in these tissues are reduced, SARS-CoV-2 infection results in changes to both the proteome and phosphoproteome. Hierarchical clustering revealed three clusters of proteins that changed expression in the heart over the course of infection. Cluster 1 showed proteins representing GO terms associated with esterase activity, heme scavenging, and binding/uptake of ligands by scavenging receptors, peaking at day 4 postinfection (see Fig. S3A in the supplemental material). The second cluster of proteins followed the kinetics of viral replication where the highest viral burdens in the heart were observed on days 2 and 4. Proteins in cluster 2 represented GO terms associated with NAD metabolism, glucose metabolism, oxidative stress, and regulation of TLR signaling (Fig. S3A). The third and final cluster decreased expression during the course of infection and was associated with metabolic processes and myeloid leukocyte-mediated immunity (Fig. S3A). The phosphoproteome showed limited changes following infection with phosphoproteins significantly changing expression that grouped into two clusters (Fig. S3C). One cluster included GO terms associated with kinase activity, myosin heavy chain binding, and translational activity. These proteins initially had increased expression that decreased on day 6 postinfection. The other cluster of phosphoproteins increased expression over the course of infection and was associated with immune responses, responses to calcium changes, and cell fate modulation (Fig. S3C). Although the viral burdens in the heart were lower substantially than those in the lungs, multiproteomic analysis revealed distinct changes to the cardiac proteome consistent with infection. We confirmed the differential expression of important cardiac effectors by immunoblotting. As shown in Fig. S3B, CAM kinase II, AKT, and phospho-AKT increased expression on days 2 and 6 postinfection. However, complement component C3d peaked expression on day 2 postinfection.

An analysis of the proteome and phosphoproteome of the kidney (see Fig. S4A and C in the supplemental material) revealed a more pronounced differential protein expression in response to infection than that of the heart (Fig. S3). As shown in Fig. S2, replicating virus was detected in the kidneys of hamsters infected i.t. with SARS-CoV-2. Similar to viral burdens in the heart, viral titers in the kidney were lower substantially than those observed in the lungs. However, proteome and phosphoproteome analysis revealed pronounced differential protein expression in response to infection. A total of 222 proteins and 93 phosphoproteins were differentially expressed. Hierarchical clustering and GO analysis of the differentially expressed proteins in the kidney showed two major clusters with proteins increasing or decreasing postinfection. A GO analysis of increased proteins showed changes in neutrophil degranulation, regulation of ERK1 and ERK2 cascade, and responses to hypoxia, whereas GO analysis of decreasing proteins showed major changes in metabolic processes, NADPH oxidation, Wnt signaling pathway, and response to hormones, among others (Fig. S4A). Phosphoproteome hierarchical clustering and GO analysis showed two distinct clusters, with one increasing through time and one decreasing (Fig. S4C). Gene ontology of increasing phosphoproteins were associated with Toll-like receptor signaling, responses to interferons, and mitochondrial changes, while the proteins with reductions in phosphorylation were associated with metabolism, metal binding, and deacetylation (see Fig. S5C in the supplemental material). All phosphoproteins and GO terms associated with the phosphoproteome of hearts and kidneys are shown in Data Set S1. Consistent with the increase in GO terms associated with neutrophil function late in infection, immunoblotting revealed a significant increase in neutrophil elastase and the kidney damage-associated protein chitinase-like 3 (Fig. S4B). Altogether, these data show that following respiratory infection of the hamster, there is dissemination to extrapulmonary tissues that impact the pathophysiology of the organ. Furthermore, our results suggest that SARS-CoV-2 infection leads to late innate immune responses, especially neutrophil responses in all target organs.

### Integrated multiproteomic analysis of lungs, hearts, and kidneys.

**FIG 10:**
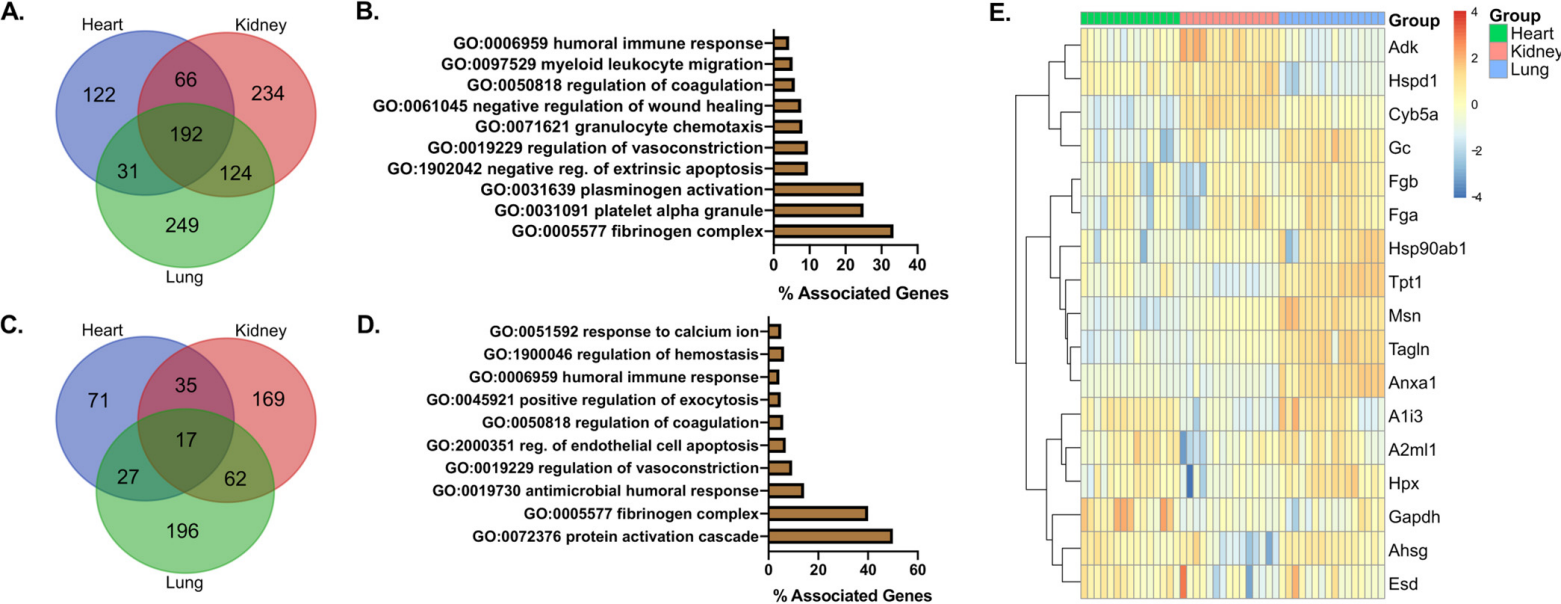
Integrated multiproteomic analysis of heart, lungs, and kidneys reveals shared factors associated with vascular injury and immune activation. (A) Venn diagram of shared proteins from global proteome analysis of lungs, hearts, and kidneys. (B) Percent associated genes of representative GO terms for 192 globally shared factors in unfiltered proteomes. (C) Venn diagram of global lung, heart, and kidney proteome factors significantly altered in at least one time point (0, 2, 4, and 6 days). (D) Percent associated genes of representative GO terms for 17 significantly changed shared factors in all tested organs. (E) Heatmap showing differential expression for the 17 significantly changed shared factors in all tested organs.

When assessing the most significantly and kinetically altered biological processes per organ individually, we observed upregulation of immune responses at day 2 postinfection. Responses to virus infection, innate and humoral responses, and cell death were the most most prevalent. At days 4 and 6, immune responses were still predominant in the lungs, while metabolic changes were observed in the hearts and kidneys. We then looked at the shared proteome in all organs. Represented in a Venn diagram, we show that 192 proteins were present via proteomic analysis in all organs (Fig. 10A). A GO analysis of shared proteomes showed terms associated with coagulation, vasoconstriction, immune cell chemotaxis, humoral immunity, and cell death (Fig. 10B). Furthermore, an analysis of significantly changed factors in all organs showed 17 proteins shared between heart, lung, and kidneys; 35 proteins were shared between heart and kidney; 27 between heart and lung; and 62 between kidney and lung (Fig. 10C). Proteins shared between all organs were found to associate with GO terms for immune system processes, cell death, vasoconstriction, humoral immune responses, calcium changes, and coagulation (Fig. 10D). A heatmap and hierarchical clustering of these 17 proteins showed their differential expression between organs demonstrating that while these factors were shared, some of them were selectively changed in a tissue-specific manner (Fig. 10E). All shared proteins and GO terms within organs are presented in Data Set S3 in the supplemental material. An analysis of shared proteins at different time points (Fig. 10) was found consistent with the transcriptomic (Fig. 3) and proteomic analysis (Fig. 6).

## DISCUSSION

SARS-CoV-2 infection ranks among the most significant public health challenges since the 1918 influenza pandemic. SARS-CoV-2 is the third human-pathogenic β-coronavirus to emerge in the last 15 years. Although significant efforts have been made to define the pathogenesis of this infection, progress has been limited by the lack of an animal model that can be naturally infected with SARS-CoV-2 while also recapitulating the serious manifestations of human disease. Here, we report a hamster model that recapitulates many of the severe manifestations of COVID-19. We investigated the impact of severe SARS-CoV-2 infection on both the transcriptome and the proteome over the course of worsening disease. These investigations revealed dynamic responses to infection that persisted beyond the window of active viral replication. A prominent type I and II antiviral response was readily apparent in the both the transcriptomic and proteomic responses to infection. Along with the antiviral response, a program of inflammatory cytokine and chemokine expression correlating with the robust infiltration of inflammatory cells was evident in both the transcriptome and proteome.

The goal of the current study was to kinetically evaluate changes in the transcriptome and proteome during the development of severe COVID-19-like disease in a hamster model. Although several small animal models of COVID-19 have been published recently, including mice, ferrets, nonhuman primates, and hamsters (12, 14, 15, 30, 33, 37), each model is limited by either not developing severe disease manifestations or requiring genetic manipulation to promote infection. Likewise, not all of the current models recapitulate some of the major features of human disease. The hamster, ferret, and nonhuman primates can be infected naturally with human isolates of SARS-CoV-2. Disease severity in these species ranges from mild to moderate or, as reported here, to severe disease. The mouse is one of the most versatile animal models for infectious disease research. However, mouse ACE2 does not promote SARS-CoV-2 invasion, and this host range restriction precludes infection of standard laboratory mice with most human clinical isolates. To circumvent this barrier to infection, a mouse expressing human ACE2 under the control of the keratin-18 promoter was originally developed to evaluate SARS-CoV infection (37). This mouse supports SARS-CoV-2 infection, and several reports suggest that severe disease develops in this mouse (33, 38, 39). However, it was noted during the original SARS studies that frequent infection of the brain occurred (37), suggesting that this humanized mouse did not mimic human disease. Infection of the brain with SARS-CoV-2 in the K18-hACE2 mouse has been reported (33, 39). Although significant neurological symptoms have been reported by COVID-19 patients, it remains unclear how SARS-CoV-2 infection impacts the brain. Other approaches for expressing human ACE2 in the mouse have been reported, including adeno-associated virus transduction (40). As the spike protein-ACE2 interaction is the major host-range determinant, mouse-adapted versions of SARS-CoV-2 with mutations in spike and other proteins have been isolated that allow infections in the mouse through uptake via mouse ACE2 (41, 42). While these approaches allow researchers to harness the power of the mouse model, they have limitations and caveats.

The hamster has been an important small animal model for a number of human infectious agents that cannot infect other animals (43). Soon after the identification of SARS-CoV-2 as the causative agent of COVID-19, it was reported that hamsters infected i.n. with clinical isolates of SARS-CoV-2 developed mild-to-moderate respiratory disease (12, 30). However, these animals do not develop disease that mimics severe COVID-19. The i.t. administration of SARS-CoV-2 to hamsters recapitulates many aspects of severe COVID-19, including viral replication in tissues, diffuse alveolar destruction, interstitial pneumonia, inflammatory cell infiltration, and death (2, 32, 44–46). Diffuse alveolar destruction is a common precursor to acute respiratory distress syndrome (47), a frequent manifestation of severe COVID-19. The presented model replicates severe human disease, for which at time of death, there is severe damage to the lungs, decreased interferon-associated responses, low-to-undetectable viral loads, and increased infiltration of proinflammatory cells (48). At the molecular level, as expected, during an acute viral infection, there is a strong IFN response with type I and II ISG signatures. Others have reported that SARS-CoV-2 infection does not induce as strong a IFN response as other respiratory viral infections (24). Interestingly, SARS-CoV is well known to manipulate IFN responses using virulence factors conserved in SARS-CoV-2 (16, 49) suggesting the robust IFN response observed in the hamster may be a blunted response. Consistent with IFN-dependent changes to both the proteome and transcriptome, an immunoblot analysis of lung tissues from infected animals demonstrates increased expression of phosphorylated IFN-stimulated transcription factors as well as ISGs. This finding is in concurrence with data published by Shen et al. demonstrating a strong ISG response in patients with severe COVID-19 (27). Currently, there is not a consensus on the magnitude of the IFN response to SARS-CoV-2, and it may be correlated with the severity of clinical disease. Analyses of lung transcriptional responses to infection with SARS-CoV-2 in the lungs of K18-hACE2 mice and mice transduced with adeno-associated virus expressing human ACE2 revealed induction of types I, II, and III IFNs along with the corresponding ISGs (33, 40). Currently, genes for type III IFN (IFN-λ/IL-28A/B) have not been identified in the Syrian hamster. Type I and III interferons induce many of the same genes, and it is possible that some of the antiviral gene and protein expression observed in the hamster is due to the actions of a currently unidentified IFN-λ.

Lee and coworkers reported that severe COVID-19 was associated with TNF-α and IL-1β signatures (26). As shown in Fig. 2G and 3, there is an induction of TNF-α and IL-1β in the hamster following SARS-CoV-2 infection. Likewise, on day 2 following infection, proteomic analysis revealed an increase in IFN-γ- and TNF-α-stimulated proteins (Fig. 3), and transcriptomic analysis showed elevation of numerous proteins known to be induced by TNF-α as well as other molecules (CXCL10, CCRL2, CCL5, CD274, DDX-58, OAS2, TNFaip3, and XDH), correlating with viral transcript expression. Proteins that inhibit the action of TNF-α or TNF-associated signaling (PTX and TNFaip3) were also strongly correlated with the presence of viral proteins in the lungs of SARS-CoV-2-infected hamsters. Many of these proteins are also induced by IFN, and it is likely that multiple signals lead to the induction of these proteins during SARS-CoV-2 infection. Altogether, these data are consistent with the cytokine signatures observed in other studies of severe COVID-19.

Cell death is a common host response to viral and bacterial infection (50–53). Microscopic examination of lung sections from SARS-CoV-2-infected hamsters revealed evidence of substantial cell death. This evidence included pyknotic nuclei, denuded epithelium, and hyaline membranes starting on day 2 and increasing through day 6 (Fig. 2A to F and not shown). GO terms associated with pyroptosis and apoptosis were represented significantly in the transcriptome and proteome through the course of infection. Indeed, validating the omics experiments, cleaved caspase-1 and −3, hallmarks of pyroptosis and apoptosis, respectively, were detected via immunoblotting of lung extracts from infected hamsters (Fig. 6). Additionally, transcripts for MLKL and RIP3K were significantly increased in lung tissues, consistent with necroptosis (54) following SARS-CoV-2 infection of the lung. Although additional work is required to dissect the mechanisms and roles of cell death in COVID-19, these data suggest that cell death is a significant feature of the host inflammatory response and tissue injury during SARS-CoV-2 infection.

While SARS-CoV-2 primarily infects the respiratory tract, extrapulmonary manifestations of COVID-19 are common (3–6, 55). Cardiac and renal complications of COVID-19 occur frequently (2, 3, 32, 56, 57). Pulmonary SARS-CoV-2 infection of hamsters results in detection of virus in the heart and kidney tissue. Viral burdens in the heart and kidneys were considerably lower than those in the lungs, but the proteome and phosphoproteome of these organs were significantly altered over the course of infection. Proteome remodeling in these tissues could be due to a systemic inflammatory reaction and or low-level viral infection (53, 58). Similar changes in the cardiac proteome and phosphoproteome have been observed only recently after pandemic influenza infection (53). Furthermore, a GO term analysis of the proteomic data suggest that infection of these organs causes infiltration of inflammatory cells and disruption of organ physiology. These data suggest that the hamster might be a useful model with which to interrogate the extrapulmonary pathologies associated with COVID-19.

Although other models, such as mice and nonhuman primates, have more established tools for mechanistic studies, the data presented here suggest that the hamster recapitulates features of severe COVID-19. The hamster shares similar pathologies, immune responses, and gene and protein expression profiles reported during human SARS-CoV-2 infection (2, 5, 59). The hamster can be infected naturally with clinical isolates of SARS-CoV-2 without additional modifications, making it an excellent tool for interrogating host-pathogen interactions during the pathogenesis of COVID-19.

## MATERIALS and METHODS

### Animals.

Male golden Syrian hamsters, of 3 to 4 weeks old, were purchased from Charles River Laboratory and used at 5 to 6 weeks of age. Animals were housed in the animal biosafety level 3 (ABSL3) laboratory at UT-Health San Antonio with a 12-h light/dark cycle and free access to food and water. Animals were sedated with ketamine (100 mg/kg) and xylazine (5 mg/kg) prior to procedures. All procedures were in accordance with approved IACUC (2020040AR and 2020048AR) and Institutional Biosafety Committee (IBC) protocols (08496).

### Virus.

SARS-CoV-2 isolate USA-WA-1/2020 was used for these studies. The reagent was deposited by the Centers for Disease Control and Prevention and obtained through BEI Resources, NIAID, NIH: SARS-Related Coronavirus 2, Isolate USA-WA-1/2020, NR-52281. The virus was expanded in Vero E6 cells; supernatants were collected and clarified by centrifugation prior to viral titers being determined via plaque assay. Passage 1 virus was used for all studies. The virus was subjected to RNA-seq analysis to characterize any potential changes to the genome as a result of expansion. These data demonstrated that the furin cleavage site in the spike was maintained and there were 13 nonsynonymous single nucleotide polymorphisms (SNPs) relative to the reference genome. Experiments with SARS-CoV-2 were done at BSL3/ABSL3 facilities.

### Plaque assays.

Titers of infectious virus were determined by plaque assay on Vero E6 cells, essentially as we have described (60). Briefly, Vero E6 cells were seeded in 6-well tissue culture plates in complete Dulbecco’s modified Eagle’s medium (DMEM) with 10% fetal bovine serum (FBS) and penicillin and streptomycin (P/S) and grown in a humidified 37°C incubator at 5% CO_2_. When cells reached 100% confluence, they were infected with 10-fold serial dilutions of virus for 1 h. Media were removed and 1.2% Avicell (Dupont) or 1.2% cellulose (catalog [cat.] no. 435244; Sigma-Aldrich) in 1× MEM with 1% FBS and P/S was added as an overlay. After 3 days, the overlay was removed, and wells were washed with phosphate-buffered saline (PBS) and fixed in 4% paraformaldehyde. Cells were then stained with 0.2% crystal violet in 20% ethanol, and plaques were revealed by washing with distilled water and subsequently enumerated.

### Animal infections.

Hamsters were infected with the indicated dose of virus either intranasally (i.n.) or intratracheally (i.t.). Hamsters infected i.n. were sedated with ketamine and xylazine; 50 μl of the virus suspension was applied to each nare, and hamsters were allowed to inhale the suspension. The i.t. infections were done essentially as we have described for mice (51); animals were sedated and the tongue was pulled forward to visualize the trachea. Three-hundred microliters of virus suspension was applied to the trachea and then the nose was covered to stimulate respiration. Animals were weighed daily and considered moribund if they lost 20% of their initial body weight as per institutional IACUC regulation. Animals were euthanized by isoflurane overdose or Euthasol (89 mg/kg) injection.

### LD_50_ determinations.

The i.t. LD_50_ was determined essentially as we have described with slight modification (61). Briefly, 3-fold serial dilutions (1 × 10^6^ to 1.2 × 10^4^) were made from the viral stock. Animals (4 hamsters/dose) were sedated and infected i.t. Infection was allowed to progress for 15 days or until animals met an euthanasia criteria.

### PFU determination from tissues.

Tissues were removed at necropsy and weighed. Tissues were then disrupted in PBS containing 1% FBS and P/S, and insoluble material was removed by centrifugation. The clarified material was frozen until used in plaque assays as described above.

### ELISA to measure cytokines.

Hamster-specific IFN-γ, IL-6, and TNF-α enzyme-linked immunosorbent assays (ELISAs) were purchased from G-Biosciences. Concentrations of cytokine in 50 μl of BALF or serum was determined according to the manufacturer’s instructions.

### Histopathology.

Tissues were collected at the indicated time points and fixed in 10% neutral-buffered formalin. Tissues were processed, blocked, cut, and stained with hematoxylin and eosin (H&E) stain by the UT Health San Antonio STRL Histopathology/Immunohistochemistry Laboratory. Images of tissue sections were obtained using a Zeiss Axioscope equipped with an HRc camera. Slides were evaluated by pathologists (Y.I. and G.H.) who were blind to treatment.

### Protein collection and mass spectrometry.

**Sample inactivation.** Following intratracheal inoculation of SARS-CoV-2, hearts, lungs, and kidneys were collected at days 0, 2, 4, and 6 with 3 to 4 animals per time point. Organs were washed; weighed; mechanically disassociated in saline containing 2× HALT protease and phosphatase inhibitors (Pierce); and inactivated in 1% SDS, 5 mM Na-EDTA, and 50 mM dithiothreitol (DTT), with heat for inactivation 95°C for 20 min.

### STrap-based protein digestion.

To digest tissue proteins, we adapted the self-packed STrap protocol in this study (62). Briefly, a Microcon filter device was disassembled and reassembled with the membrane replaced with two layers of Whatman GF/F membrane cut into 10/32″ discs. The filter device was placed in a collection tube, and membrane discs were flushed with 400 μl binding buffer (90% methanol and 100 mM triethylammonium bicarbonate [TEAB] [pH 7.1]) by centrifuging at 2,000 rpm for 1 to 2 min. For global proteome analysis, ∼50 μg protein from tissue homogenates generated with SDS was incubated at 95°C for 10 min with 20 mM DTT. Protein samples were cooled to room temperature, and iodoacetamide (IAA) was added to a final concentration of 55 mM. Samples were incubated in the dark for 30 min. Phosphoric acid was added to a final concentration of 1.2%, and samples were gently mixed for 1 to 2 min. Six volumes of binding buffer was added to the protein samples and incubated for 1 to 2 min with gentle mixing. Samples were loaded onto the STrap filter device and spun at 2,000 rpm for 1 to 2 min until the solution was transferred to the bottom collection tube. Sample loading was repeated until all the solution passed though the filter while the protein suspension remained on top of the filter. The protein particulate was washed with 400 μl binding buffer and centrifuged. The wash step was repeated three times. The filter device was transferred to a new collection tube, and 150 μl digestion buffer (50 mM TEAB in water) with trypsin (trypsin:protein [wt/wt], 1:50) was added. The digestion was incubated overnight at 37°C without shaking. To elute peptides, sequential elution with three elution buffers was performed (50 mM TEAB, 0.2% formic acid [FA], and 0.2% FA/50% acetonitrile [ACN]; 200 μl each). Eluents were pooled and dried in a SpeedVac instrument. After we desalted using C_18_-based StageTip (63), the peptides were ready for LC-MS/MS analysis.

### Phosphopeptide enrichment.

For the enrichment of phosphopeptides, a StageTip-based protocol was followed (64). Approximately 0.6 mg of TiO_2_ beads were used per 100 μg of peptides. Briefly, TiO_2_ beads (GL Science, Torrance, CA) were first resuspended in 100% ACN and packed into the tip with one layer of C_8_ membrane as frit. The tip was centrifuged at 3,000 × *g* for 1 to 2 min to remove ACN. A total of 400 to 500 μg of peptides from the protein digest was resuspended with loading buffer (60% acetonitrile and 5% trifluoroacetic acid [TFA]), sonicated for 3 min (pulse: 30 sec on, 30 sec off), and centrifuged at highest speed for 10 min to pellet insoluble particles. The supernatant was then loaded onto the TiO_2_ tip and centrifuged at 500 × *g* for 6 to 10 min until all the solution passed the beads. The beads were washed 2 times with 200 μl wash buffer (80% ACN and 2% TFA) and centrifuged at 3,000 × *g* for 1 to 2 min. Phosphopeptides were eluted with 90-μl elution buffer (40% ACN and 15% NH_4_OH) and collected by centrifugation at 500 × *g* for 6 to 10 min. Peptides were neutralized with 9 μl formic acid and desalted by C_18_-based StageTip as described previously (63) before LC-MS/MS analysis.

### LC-MS/MS analysis.

The LC-MS/MS analysis was carried out using an Ultimate 3000 nanoLC system coupled to Q Exactive mass spectrometer (Thermo Scientific). Peptides were first loaded onto a trap column (PepMap C_18_; 2 cm by 100 mm internal diameter [I.D.]; Thermo Scientific) and then separated by an in-house-packed analytical column (C_18_ ReproSil, 3.0 mm; Maisch GmbH; 20 cm by 75 mm I.D.) using a binary buffer system (buffer A, 0.1% formic acid in water; buffer B, 0.1% formic acid in acetonitrile) with a 150-min gradient (2% to 35% buffer B over 105 min; 35% to 80% buffer B over 10 min; back to 2% B in 5 min for equilibration after staying on 80% B for 5 min). Two LC-MS runs (technical replicates) were performed for each of the three or four biological replicate samples essentially as we have described (53). MS data were acquired in a data-dependent top-10 method with a maximum injection time of 20 ms, a scan range of 350 to 1,800 Da, and an automatic gain control (AGC) target of 1e6. MS/MS was performed via higher energy collisional dissociation fragmentation with a target value of 5e5 and maximum injection time of 100 ms. Full MS and MS/MS scans were acquired at resolutions of 70,000 and 17,500, respectively. Dynamic exclusion was set to 20 s.

### Database search and bioinformatics analysis.

Protein identification and quantitation were performed using the MaxQuant-Andromeda software suite (version 1.6.5.0) with most of the default parameters (65). A Mesocricetus auratus (golden Syrian hamster) database (NCBI: txid10036) was used for the database search. For the global proteome analysis, the following parameters were applied: 4.5 ppm and 20 ppm mass tolerances for precursor and fragments, respectively; trypsin as an enzyme that maximally allowed two missed cleavage sites; protein N-terminal acetylation and methionine oxidation as variable modifications; cysteine carbamidomethylation as a fixed modification; and peptide length was at least 7 amino acids. For phosphoproteome analysis, phosphorylation at serine, threonine, and tyrosine was set as an additional variable modification. False discovery rate was set at 1% for both proteins and peptides. The cutoff of phosphosite probability estimated by MaxQuant was required to be 0.75 or higher. Proteins which are absent in more than 50% of samples either in T0, T2, T4, or T6 groups were removed from the data. Missing values were imputed using the reference 66 algorithm with the LSimpute_array option. For global quantitative proteome analysis, the technical replicates (e.g., different LC-MS runs) were merged during MaxQuant analysis.

### Immunoblots.

Samples for Western blotting were homogenized in PBS and sonicated and then diluted in Laemmli buffer and aliquoted for storage at −80°C. Samples were loaded into 4% to 15% gradient gels at 20 μg per lane, separated by SDS-PAGE, and transferred to nitrocellulose membranes. Total protein was quantified by Ponceau stain, and then membranes were blocked in blocking buffer (Tris-buffered saline [TBS]-0.01% Tween 20 containing 5% bovine serum albumin [BSA]) for at least 1 h. Primary antibodies were diluted in blocking buffer, and membranes were incubated overnight at 4°C. Primary antibodies used included the following: gasdermin D (1:1,000; Cell Signaling; catalog no. 93709S), caspase-1 (1:1,000; Cell Signaling; 24232S), cleaved caspase-3 (1:500; Cell Signaling; 9661T), b-actin (1:1,000; ProteinTech; 60008-1-AP), CaMKII pT286 (1:1,000; Cell Signaling; 127116T), pan-AKT (1:1,000; Cell Signaling; 4691T), AKT pS473 (1:1,000; Cell Signaling; 9271T), GST (1:1,000; Cell Signaling; 2622), catalase (1:1,000; ProteinTech; 21260-1-AP), C3 (1:1,000; ProteinTech; 21337-1-AP), neutrophil elastase (1:1,000; Abcam; ab68672), myeloperoxidase (1:1,000; Abcam; ab208670), ACE2 (1:1,000; ProteinTech; 21115-1-AP), ISG-15 (1:30,000; ProteinTech; 15981-1-AP), IRF3 (1:30,000; Cell Signaling; 11904S), IRF3 pS386 (1:30,000; Cell Signaling; 37829S), IRF7 (1:30,000; Cell Signaling; 13014S), IRF7 pS477 (1:30,000; Cell Signaling; 12390S), STAT1 (1:30,000; Cell Signaling; 9172S), STAT1 pY701 (1:30,000; Cell Signaling; 9167S), STAT3 (1:30,000; Millipore; 07-2173), STAT3 pY705 (1:30,000; Santa Cruz; SC-8059), SLAMF9 (1:30,000; R&D Systems; MAB2829), RSAD2 (1:30,000; Sigma; AV4696), GAPDH (1:30,000; MA5-15738), and nucleocapsid (1:30,000; GeneTex; GTX135357). Horseradish peroxidase (HRP)-tagged secondary antibodies were used to detect primary antibodies (1:10,000 in blocking buffer) for 1 h. SuperSignal West PICO Plus (ThermoFisher; 34580) was used to develop HRP. All images were collected on an Amersham Imager 680 (GE) and analyzed for densitometry in ImageJ. Briefly, to relatively quantify protein bands from Western blots relative to loading control (here, total loaded protein), after images were collected, the picture mode was changes to “grayscale.” Under the “analyze” menu, measurements were set to read gray mean value. The regions to be analyzed were selected with the selection tool, and also a background read was done by reading an area of the gel without antibody-stained bands. After the net bands and loading controls were calculated as the final step, a ratio of a net band value over the net loading control of that lane was recorded.

### RNA sequencing.

Total RNA was isolated from the lungs of hamsters infected with SARS-CoV-2 or mock treated with vehicle at the indicated time. Lungs were homogenized, placed in TRIzol, and frozen until used. Bulk RNA in TRIzol was purified according to the manufacturer’s instructions. RNA was further purified using the RNeasy kit (Qiagen). Library preparation and RNA-seq was performed by the Genome Sequencing Facility at the Greehey Children’s Cancer Institute, UT Health San Antonio. The RSEM package along with Bowtie2 were used for quantifying gene and isoform abundances from paired-end RNA-seq data (67). Differential gene expression analysis was performed using the edgeR package (68).

### Statistical analysis.

Data were analyzed statistically using the R or GraphPad Prism 8 suites of software. Differential analysis for proteomic data was performed using the Limma software package in R (69). Data are presented as the standard error of the mean (SEM) and are representative of a minimum of two independent experiments. Data points for quantitative *in vitro* experiments represent all technical repeats for experiments done in triplicate. The number of animals used in *in vivo* experiments is indicated in the figure legend. Survival curves were analyzed by log-rank (Mantel-Cox test). Other analyses of large data sets were analyzed as indicated below.

### Gene ontology, clustering, and classification analyses.

Gene ontology information of RNA transcripts and proteins was obtained using the ClueGO (70) plug-in in Cytoscape software (71). We considered the biological process, cellular component, molecular function, and immune system process ontologies with the “all evidence” option and kept the number of clusters at 2 under the advance term/pathway selection option. The heatmaps were created using the pheatmap package in R using Euclidean distance matrix and ward.D as the clustering option. The volcano plots were generated using the EnhancedVolcano package in R.

### Data availability.

The data that support the findings of this study are available from the corresponding author upon reasonable request. The mass spectrometry raw data related to lung, heart, and kidney proteomics and phosphoproteomics have been deposited in the MassIVE repository under the MassIVE accession number MSV000086925 (https://doi.org/10.25345/C5BN4P) and doi number 10.25345/C5BN4P. Transcriptomic data have been made publicly available at BioProject with data identifier PRJNA704997.
