# Supplementary material for: Kinetic Multi-omic Analysis of Responses to SARS-CoV-2 Infection in a Model of Severe COVID-19
Source: J Virol. 2021 Sep 27;95(20):e01010-21. doi: 10.1128/JVI.01010-21 (PMC8475517; doi:10.1128/JVI.01010-21)
Supplement: Supplemental file 4 — Fig. S1 to S5; legends to Data Sets S1 to S3. Download JVI.01010-21-s0001.pdf, PDF file, 2.7 MB [file jvi.01010-21-s0001.pdf]

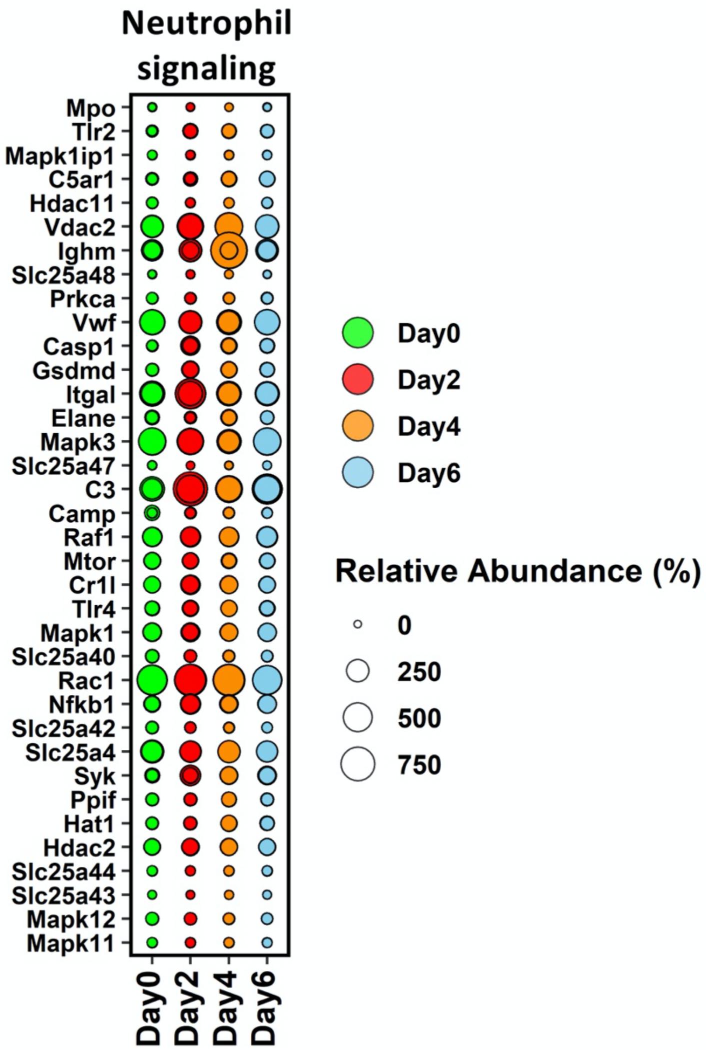

Supplemental Figure 1: Severe SARS-CoV-2 infection leads to differential expression of transcripts associated with neutrophil activity. Bubble plot of significantly changed transcripts associated with neutrophil activation and neutrophil extracellular trap formation. Bubble size denotes relative abundance and color shows time points: green (day 0, uninfected), red (day 2), orange (day 4) and blue (day 6).

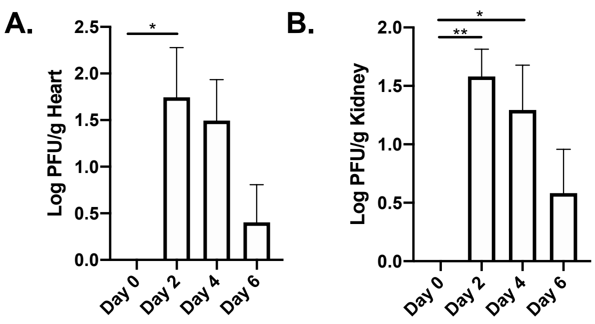

Supplemental Figure 2: Viral titers in hearts and kidneys post intratracheal challenge of golden Syrian hamsters. Male 5-to-6-week-old golden Syrian hamsters were intratracheally infected with SARS-CoV-2 strain USA-WA-1/2020 or mock challenged with vehicle (DMEM) and lungs excised at days 2, 4 and 6 post-infection. Log PFU per gram of tissue of (**A**) hearts and (**B**) kidneys assessed via plaque assays.

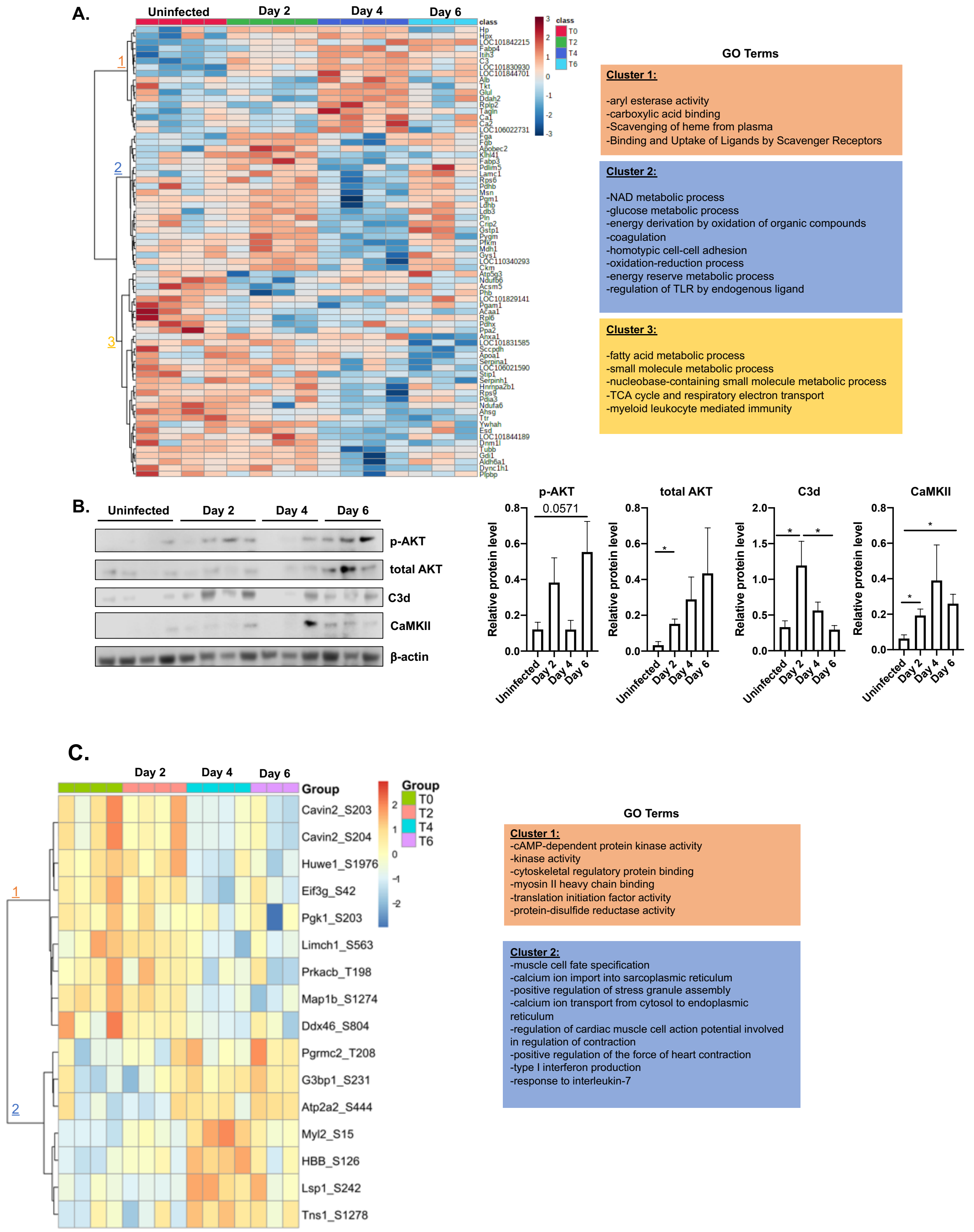

A.
Uninfected
Day 2
Day 4
Day 6
class
T0
T2
T4
T6
1
2
3
GO Terms
Cluster 1:
-aryl esterase activity
-carboxylic acid binding
-Scavenging of heme from plasma
-Binding and Uptake of Ligands by Scavenger Receptors
Cluster 2:
-NAD metabolic process
-glucose metabolic process
-energy derivation by oxidation of organic compounds
-coagulation
-homotypic cell-cell adhesion
-oxidation-reduction process
-energy reserve metabolic process
-regulation of TLR by endogenous ligand
Cluster 3:
-fatty acid metabolic process
-small molecule metabolic process
-nucleobase-containing small molecule metabolic process
-TCA cycle and respiratory electron transport
-myeloid leukocyte mediated immunity
B.
Uninfected
Day 2
Day 4
Day 6
p-AKT
total AKT
C3d
CaMKII
β-actin
p-AKT
total AKT
C3d
CaMKII
0.0571
Relative protein level
Uninfected
Day 2
Day 4
Day 6
C.
Day 2
Day 4
Day 6
Group
T0
T2
T4
T6
Cavin2_S203
Cavin2_S204
Huwe1_S1976
Eif3g_S42
Pgk1_S203
Limch1_S563
Prkacb_T198
Map1b_S1274
Ddx46_S804
Pgrmc2_T208
G3bp1_S231
Atp2a2_S444
Myl2_S15
HBB_S126
Lsp1_S242
Tns1_S1278
1
2
GO Terms
Cluster 1:
-cAMP-dependent protein kinase activity
-kinase activity
-cytoskeletal regulatory protein binding
-myosin II heavy chain binding
-translation initiation factor activity
-protein-disulfide reductase activity
Cluster 2:
-muscle cell fate specification
-calcium ion import into sarcoplasmic reticulum
-positive regulation of stress granule assembly
-calcium ion transport from cytosol to endoplasmic reticulum
-regulation of cardiac muscle cell action potential involved in regulation of contraction
-positive regulation of the force of heart contraction
-type I interferon production
-response to interleukin-7

Supplemental Figure 3: Severe SARS-CoV-2 leads to proteomic remodeling of hamster hearts. Male 5-to-6-week-old golden Syrian hamsters were intratracheally infected with SARS-CoV-2 strain USA-WA-1/2020 or mock challenged with vehicle (DMEM) and hearts excised at days 2, 4 and 6 post-infection. (**A**) Proteomic changes of hamster lungs after SARS-CoV-2 infection or mock challenge (n = 3-4 per timepoint). Hierarchical clustering of LFQ intensities of significantly changed proteins (ANOVA, FDR < 0.01) revealed three distinct clusters. Their abundance profiles among the groups were plotted in the heatmap. Enriched GO biological process terms are indicated for each marked cluster. (**B**) Immunoblots for complement C3d, AKT, phospho-AKT, CamKII and actin as loading control (n = 3-4 per group). Histograms of protein level quantification (densitometry). Kruskal-Wallis test with Dunn's multiple-comparison post-test. Asterisks denote the level of significance observed: * = $p \leq 0.05$. (**C**) Heatmap of phosphorylation changes of hamster kidney proteins after SARS-CoV-2 infection or mock challenge (n = 3-4 per timepoint). Hierarchical clustering of proteins with significantly changed phosphorylation state (ANOVA) revealed 2 distinct major clusters. Enriched GO biological process terms for each cluster are indicated.

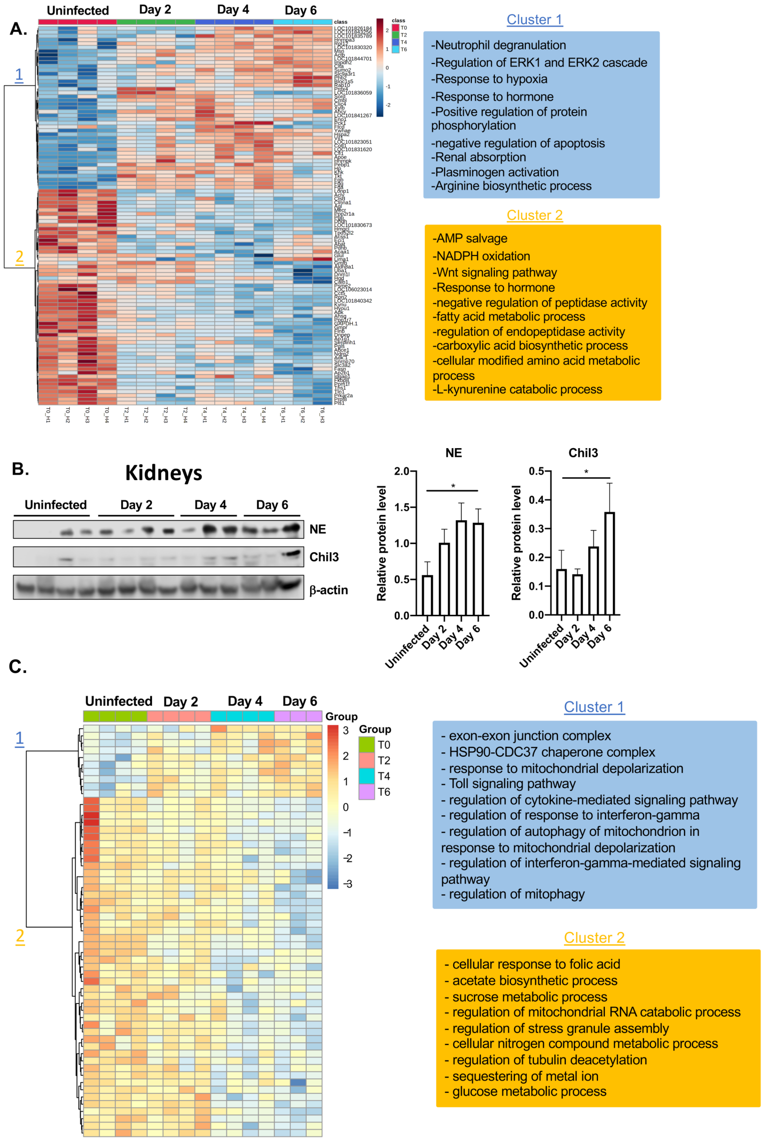

Supplemental Figure 4: SARS-CoV-2 infection leads to proteomic changes associated with tissue injury. Male 5-to-6-week-old golden Syrian hamsters were intratracheally infected with SARS-CoV-2 strain USA-WA-1/2020 or mock challenged with vehicle (DMEM) and kidneys excised at days 2, 4 and 6 post-infection. (**A**) Proteomic changes of hamster kidneys after SARS-CoV-2 infection or mock challenge (n = 3-4 per timepoint). Hierarchical clustering of LFQ intensities of significantly changed proteins (ANOVA, FDR < 0.01) revealed two distinct clusters. Their

abundance profiles among the groups were plotted in the heatmap. Enriched GO biological process terms are indicated for each marked cluster. (**B**) Immunoblots for neutrophil elastase (NE), chitinase 3 (Chil3) and actin as loading control (n = 3-4 per group). Histograms of protein level quantification (densitometry). Kruskal-Wallis test with Dunn's multiple-comparison post-test. Asterisks denote the level of significance observed: * = $p \leq 0.05$. (**C**) Phosphoproteome analysis of kidneys after SARS-CoV-2 infection reveals temporal changes to mitochondrial health and immune responses. Male 5-to-6-week-old golden Syrian hamsters were intratracheally infected with SARS-CoV-2 strain USA-WA-1/2020 or mock challenged with vehicle (PBS) and kidneys excised at days 2, 4 and 6 post-infection.

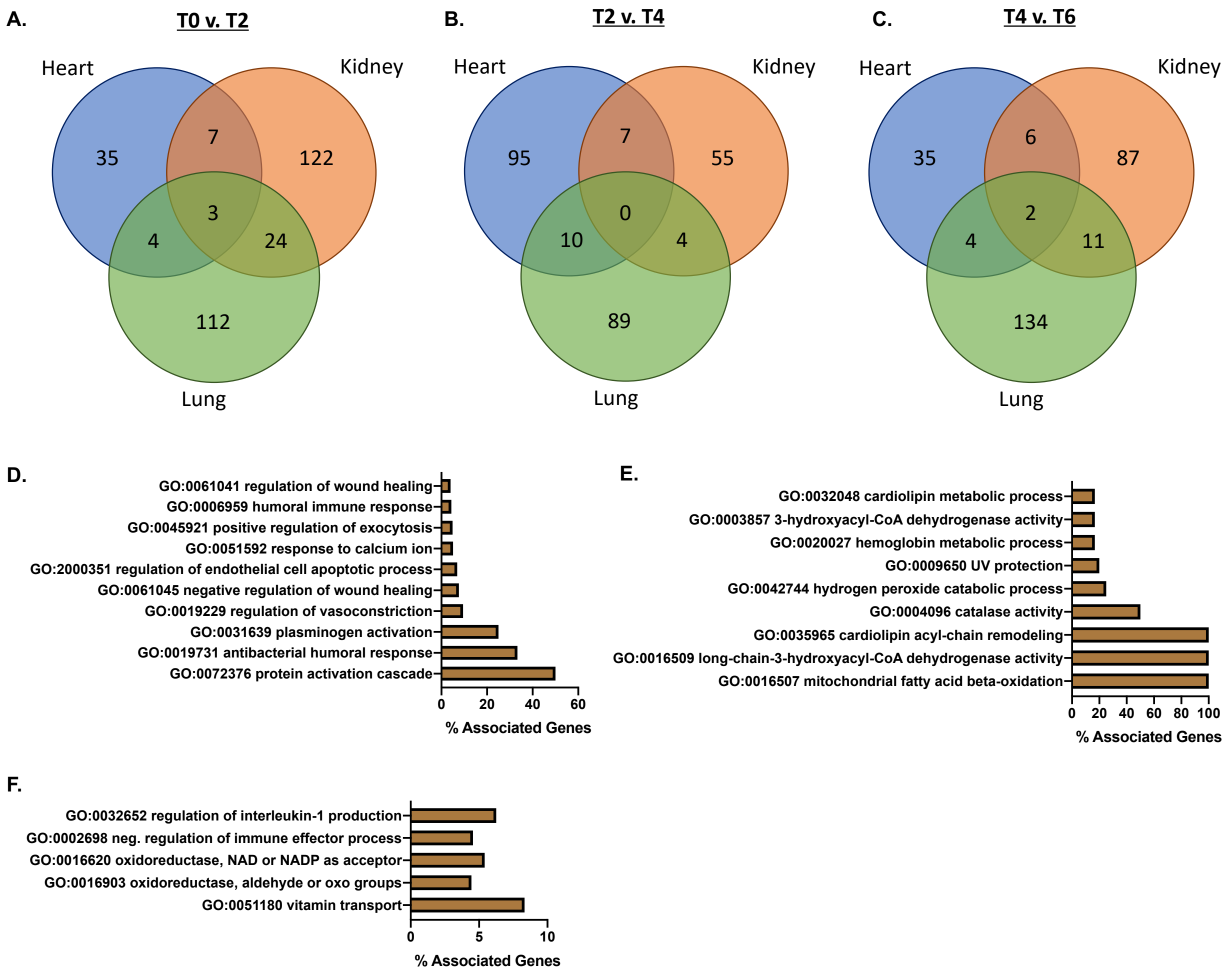

Supplemental Figure 5: Integrated kinetic multi-proteomic analysis of heart, lungs and kidneys. Venn diagram of shared proteins from global proteome analysis of lungs, hearts and kidneys at comparisons within times (**A**) T0/T2, (**B**) T2/T4 and (**C**) T4/T6. % associated genes of representative GO terms for (**D**) heart/lung/kidney at T0/T2, (**E**) heart/lung at T2/T4 and (**F**) heart/kidney/lung at T4/T6 shared factors.

Supplemental Data Set 1.

All phosphoproteins and GO terms associated with the phosphoproteome are shown.

Supplemental Data Set 2.

Full GO-term list with associated proteins and transcripts from the integrated omic analysis.

Supplemental Data Set 3.

All shared proteins and GO terms within lungs, hearts, and kidneys are presented.
